# *M. tuberculosis* Reprograms Hematopoietic Stem Cells to Limit Myelopoiesis and Impair Trained Immunity

**DOI:** 10.1016/j.cell.2020.09.062

**Published:** 2020-10-29

**Authors:** Nargis Khan, Jeffrey Downey, Joaquin Sanz, Eva Kaufmann, Birte Blankenhaus, Alain Pacis, Erwan Pernet, Eisha Ahmed, Silvia Cardoso, Anastasia Nijnik, Bruce Mazer, Christopher Sassetti, Marcel A. Behr, Miguel P. Soares, Luis B. Barreiro, Maziar Divangahi

**Affiliations:** 1 Meakins-Christie Laboratories, Department of Medicine, Department of Microbiology and Immunology, Department of Pathology, McGill University, Montreal, QC, Canada; 2 McGill International TB Centre, McGill University Health Centre, Montreal, QC, Canada; 3 Department of Theoretical Physics, University of Zaragoza, Institute BIFI for Bio-computation and Physics of Complex Systems, University of Zaragoza, Zaragoza, Spain; 4 Instituto Gulbenkian de Ciencia, Oeiras, Portugal; 5 Department of Medicine, Genetic Section, University of Chicago, Chicago, IL, USA; 6 Department of Physiology, Complex Traits Group, McGill University, Montreal, QC, Canada; 7 Department of Microbiology and Physiological Systems, University of Massachusetts Medical School, Worcester, MA, USA

**Keywords:** trained immunity, hematopoietic stem cells, macrophages, monocytes, iron metabolism, type I IFN, myelopoiesis, necroptosis, Mycobacterium tuberculosis, BCG

## Abstract

A greater understanding of hematopoietic stem cell (HSC) regulation is required for dissecting protective versus detrimental immunity to pathogens that cause chronic infections such as *Mycobacterium tuberculosis* (*Mtb*). We have shown that systemic administration of Bacille Calmette-Guérin (BCG) or β-glucan reprograms HSCs in the bone marrow (BM) via a type II interferon (IFN-II) or interleukin-1 (IL1) response, respectively, which confers protective trained immunity against *Mtb*. Here, we demonstrate that, unlike BCG or β-glucan, *Mtb* reprograms HSCs via an IFN-I response that suppresses myelopoiesis and impairs development of protective trained immunity to *Mtb*. Mechanistically, IFN-I signaling dysregulates iron metabolism, depolarizes mitochondrial membrane potential, and induces cell death specifically in myeloid progenitors. Additionally, activation of the IFN-I/iron axis in HSCs impairs trained immunity to *Mtb* infection. These results identify an unanticipated immune evasion strategy of *Mtb* in the BM that controls the magnitude and intrinsic anti-microbial capacity of innate immunity to infection.

## Highlights

• BCG and *Mtb* uniquely reprogram HSCs for at least 1 year
• *Mtb* suppresses myelopoiesis and impairs HSC engraftment
• *Mtb* hijacks a type I IFN/iron axis to induce necroptosis specifically in myeloid lineage
• Reprogramming of HSCs by a type I IFN/iron axis impairs trained immunity

## Graphical Abstract

**Figure:**
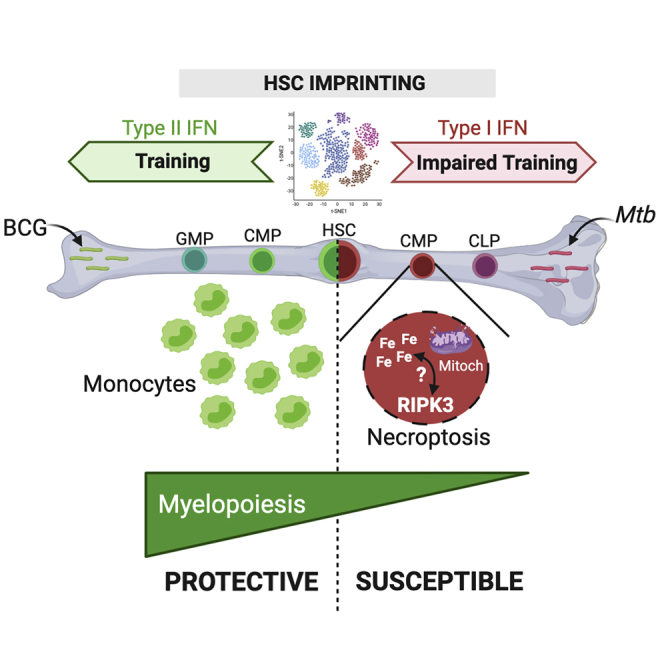

## Teaser

The pathogen *Mycobacterium tuberculosis* reprograms hematopoietic stem cells and limits myelopoiesis while impairing trained immunity responses via a type I IFN/iron signaling axis.

## Introduction

During infection or stress, hematopoietic stem cells (HSCs) interrupt dormancy and adapt to meet the peripheral demand for immune cells via their expansion and differentiation into more lineage-restricted progenitors, primarily within the bone marrow (BM). This process must be tightly controlled to avoid dysregulated HSC activation, which may lead to exhaustion and compromise the systemic immune compartment (Essers et al., 2009; Hartner et al., 2009; Sato et al., 2009). Infection-specific changes in hematopoiesis may restrict or promote generation of specific lineages to centrally bias the overall systemic immune response. For instance, we recently demonstrated that exposure of BM HSCs to Bacille Calmette-Guérin (BCG) or β-glucan (a component of the fungal cell membrane) results in their reprogramming to promote myelopoiesis and protect against *Mycobacterium tuberculosis* (*Mtb*) infection in a type II interferon (IFN-II)- or interleukin-1 (IL1)-dependent manner, respectively (Kaufmann et al., 2018; Moorlag et al., 2020). Thus, safeguard mechanisms must exist to ensure maintenance and survival of the HSC pool and their commitment toward myelopoiesis.

Although initially confined to cells of the lymphoid lineage, memory-like responses have now been described extensively for myeloid cells in a process called trained immunity (Netea et al., 2020). The short lifespan of innate immune cells, however, limits the effectiveness of this protective response against chronic infections. Others (Mitroulis et al., 2018) and we (Kaufmann et al., 2018; Moorlag et al., 2020) have demonstrated that trained immunity is driven by HSC epigenetic imprinting, which can be transmitted to BM-derived macrophages. Thus, imprinting of innate memory signatures in HSCs appears to overcome the limitation of the short half-life mature innate cells. Although protective trained immunity is well documented, it may also manifest deleteriously. For example, the persistent hyperactive state of trained innate cells can trigger tissue damage or chronic inflammation upon exposure to endogenous ligands (Bekkering et al., 2014; Leentjens et al., 2018) or pathogen-associated molecular patterns (PAMPs) (Wendeln et al., 2018), suggesting that the stimulus, localization, and cell type are crucial for promoting development of protective versus detrimental rewiring. Although exposure of HSCs to certain pathogens and/or PAMPs appears to be an important step for generation of protective trained immunity, the mechanistic basis of this process as well as its longevity remain largely unknown.

IFN-I (IFN-α and IFN-β) and IFN-II (IFN-γ) are critical for regulation of HSC activation (Baldridge et al., 2010; Essers et al., 2009) and are also involved in generation of epigenetically mediated innate memory responses (Kamada et al., 2018). Although chronic exposure of HSCs to IFN-I (Sato et al., 2009) or IFN-II (Matatall et al., 2016) causes exhaustion, IFN-II signaling is also required for HSC proliferation at steady state (Baldridge et al., 2010) and promotion of myelopoiesis in systemic BCG vaccination (Kaufmann et al., 2018) or malaria infection (Belyaev et al., 2010). Although these cytokines are involved in regulating survival and expansion of progenitors, they do not appear to directly induce HSC lineage commitment. Rather, cell death appears to be a key mechanism by which HSC and progenitor populations promote lineage biasing. For instance, during bacterial infection, IFN-I signaling inhibits HSC expansion via necroptosis (Smith et al., 2018), whereas overexpression of the anti-apoptotic proteins BcL-xL (Dolznig et al., 2002) and Bcl-2 (Akashi et al., 1997) rescues erythropoiesis in erythropoietin-deficient mice and lymphopoiesis in IL-7R-deficient mice, respectively. However, the molecular mechanisms involved in HSC fate decisions are incompletely understood.

Iron (Fe) is a vital micronutrient that supports fundamental cellular functions in most living organisms, where regulation of Fe metabolism emerged evolutionarily to form a conserved host defense strategy (Hood and Skaar, 2012; Soares and Weiss, 2015). During chronic *Mtb* infection, the host minimizes Fe availability to inhibit bacterial growth (Olakanmi et al., 2007; Parrow et al., 2013), and major alterations in Fe metabolism lead to susceptibility to tuberculosis (TB) (Gangaidzo et al., 2001; Murray et al., 1978). Yet, the potential contribution of Fe to HSC lineage decisions during infection is unknown.

Considering that BCG reprograms HSCs toward myelopoiesis and generates protective trained immunity against *Mtb* (Cirovic et al., 2020; Kaufmann et al., 2018), we investigated whether the presence of virulent *Mtb* in the BM affects innate immunity to infection. Here we show that, although access of *Mtb* to the BM changes the transcriptional landscape of HSCs and multipotent progenitors (MPPs) similarly to BCG, the magnitude of the IFN-I and heme metabolism pathways significantly differed between BCG and *Mtb*. In contrast to BCG, *Mtb* induced RIPK3-dependent necroptosis in myeloid progenitors downstream of HSCs via an IFN-I/Fe axis, which led to reduced myelopoiesis and failure to generate trained immunity against TB. Importantly, the protective or detrimental signatures of BCG and *Mtb* on HSCs were maintained for ∼1 year, respectively. Thus, our study indicates that *Mtb* accesses the BM to target innate immunity by imprinting HSCs with a unique transcriptomic profile that suppresses myelopoiesis and impairs innate immune control of *Mtb* infection.

## Results

### Systemic *Mtb* Differentially Modulates Hematopoiesis Compared with BCG in a Region of Difference 1 (RD1)-Dependent Manner

**Figure 1:**
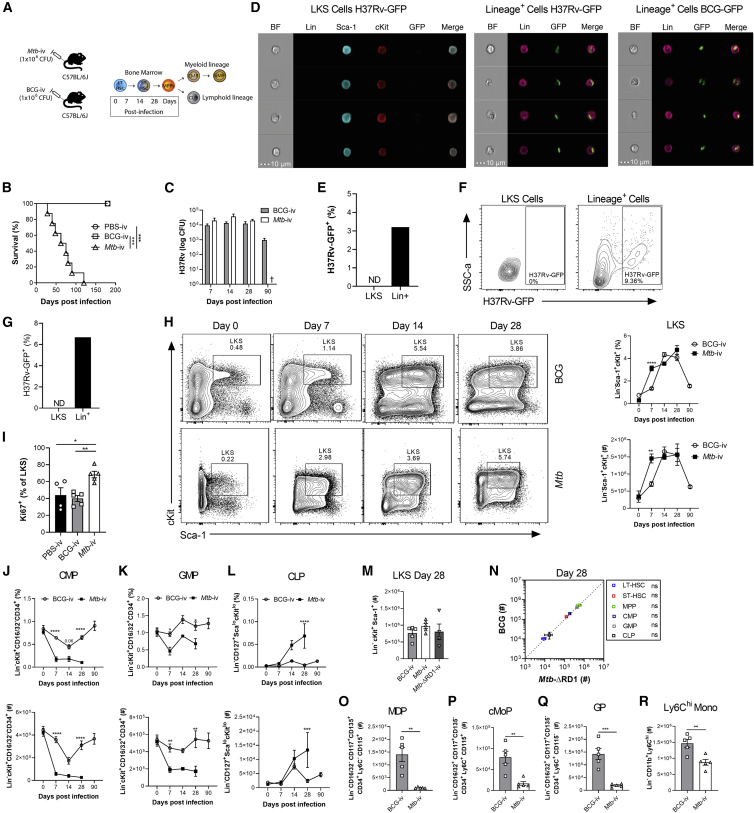
*Mtb*-i.v. Induces HSC Expansion and Suppresses Myelopoiesis (A) The i.v. model. (B, C, and H–R) WT mice were challenged i.v. with *Mtb*, *Mtb*-ΔRD1, or BCG. (B) Survival (n = 6–8 mice/group). (C) BM CFUs (n = 4–10 mice/group). (D–G) *In vitro* infection of BM cells with H37Rv- or BCG-GFP (4 h, MOI 3). Shown are ImageStream (D) and flow cytometry (F) analysis of infected cells, as a percentage of parental cells in (E) and (G). (H) Fluorescence-activated cell sorting (FACS) plots and quantification of LKS cells (n = 4–8 mice/group). (I) Percentage of Ki67^+^ LKS cells on day 7. (J–L) Frequencies (top) and totals (bottom) of CMPs (J), GMPs (K), and CLPs (L) (n = 4–13 mice/group). (M–R) Total BM LKS cells (M); LT-HSCs, ST-HSCs, MPPs, CMPs, GMPs, and CLPs (N); MDPs (O); cMoPs (P); GPs (Q); and Ly6C^hi^ monocytes (R) on day 28. Log-rank test (B), two-way ANOVA followed by Sidak’s multiple comparisons test (C, H, and J–L), one-way ANOVA followed by Tukey’s multiple comparisons test (I and M), and two-tailed Student’s t test (N–R) were used. Data are representative of two (C, I, and O–R) or three (H and J–N) independent experiments. See also Figure S1.

Although it has been shown previously that *Mtb* can disseminate to the BM in diverse TB patients (Das et al., 2013), the effect of *Mtb* in the BM in comparison with BCG on HSC proliferation, fate decision, trained immunity, and disease pathogenesis remains unknown. To investigate this, we began by determining the effects of the same dose (1 × 10^6^ colony-forming units [CFUs]) of intravenous BCG and *Mtb* (H37Rv) (Figure 1A) on survival, BM bacterial loads, and hematopoietic cell responses in wild-type (WT) mice. Although all intravenous BCG (BCG-i.v.) and control mice survived, *Mtb*-i.v. mice succumbed to infection by 120 days (Figure 1B). *Mtb*-i.v. rapidly accessed the BM and persisted until at least 28 days post-infection, coinciding with the earliest onset of mortality. At these time points, BCG and *Mtb* entered the BM and replicated similarly (Figure 1C). As shown previously with BCG-GFP (Kaufmann et al., 2018), *Mtb* (H37Rv-GFP) was unable to infect Lin^−^ c-Kit^+^ Sca-1^+^ (LKS) cells, whereas Lin^+^ cells were readily infected, as evidenced by ImageStream (Figures 1D and 1E) and flow cytometry (Figures 1F and 1G). Thus, any effect of these mycobacteria on HSCs must be indirect.

**Figure S1:**
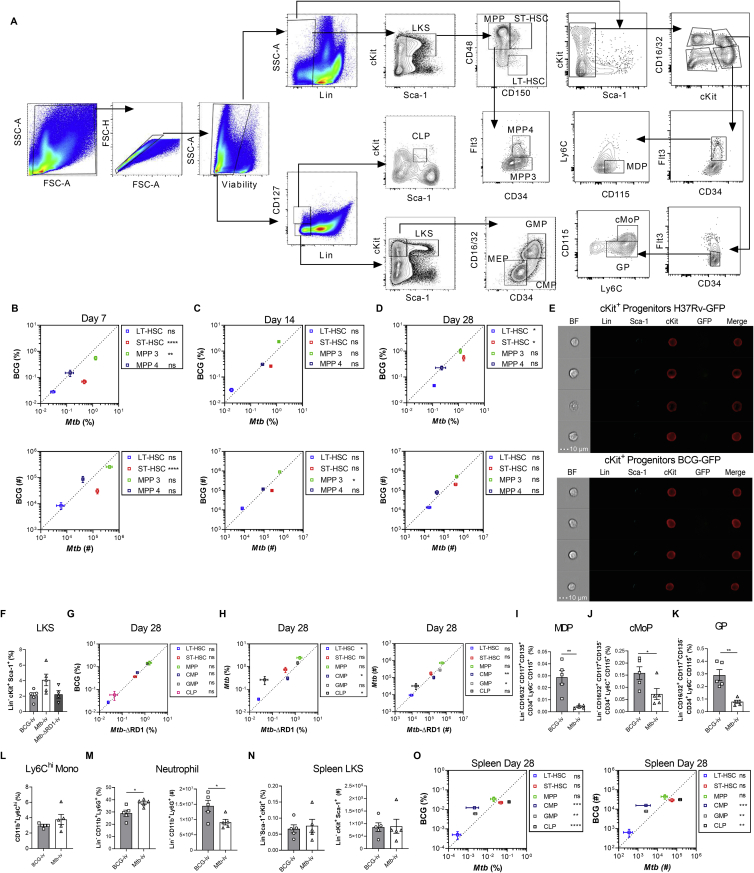
Gating Strategy for HSCs and Progenitors and Their Kinetics Post-infection, Related to Figure 1 (A) Cells were gated for FSC-A against SSC-A. Doublets were excluded using FSC-H against FSC-A. Viable cells were gated and lineage-committed cells were excluded. Within the lineage-negative population, cells were gated as LKS-defined as double positive for cKit and Sca-1. Gated on the LKS population, cells were divided into LT-HSC, ST-HSC and MPP based on CD150 and CD48 expression. MPPs were characterized as MPP3 or MPP4 by their surface expression of CD34 and Flt3. In a second strategy, lineage negative cells were gated based on CD127^+^ and CD127^-^. Lin^-^ CD127^-^ population was further defined by Sca-1 and c-Kit. c-Kit^+^ Sca-1^-^ cells were further gated based on CD34 and CD16/32 to define CMP, GMP and MEP. Lineage^-^ and CD127^+^ cells are defined as CLPs based on Sca-1^lo^ and c-Kit^lo^ expression. Finally, in another set of experiments, Lineage^+^ cells and then Sca-1^+^ cells were excluded. The remaining cells were further subdivided into cKit^+^ CD16/32^+^ and cKit^+^ CD16/32^-^ groups. In the cKit^+^ CD16/32^-^ group, CD34^+^ Flt3^+^ cells were denoted as MDP by being CD115^+^ but Ly6C^-^. cKit^+^CD16/32^+^ cells were further gated on CD34^+^ Flt3^-^ cells. Within this fraction, Ly6C^+^ CD115^-^ cells were the GP and Ly6C^+^ CD115^+^ were cMoP. (B-D) Kinetics of the frequency among single viable BM cells (top panel) and total cell counts (bottom panel) of LT-HSC, ST-HSC, MPP3/MPP4 in the BM of BCG-iv vaccinated or *Mtb*-iv infected mice. (E) BM cells from WT mice were infected with BCG-GFP or H37Rv-GFP for 4 hours *in vitro* (MOI 3). ImageStream analysis of H37Rv-GFP infection (top panel) and BCG-GFP (bottom panel) in Lin^-^ cKit^+^ Sca-1^-^ progenitors. (F-H) Mice were intravenously infected with 1x10^6^ CFU of BCG, *Mtb* or *Mtb*-ΔRD-1 for 28 days. Frequency of LKS in each group (F) and HSC/progenitor subsets of BCG versus *Mtb*-ΔRD-1 (G), or *Mtb* versus *Mtb*-ΔRD-1 (H; left panel frequency, right panel total cell counts). (I-O) 1x10^6^ CFU of BCG or *Mtb* were delivered intravenously for 28 days. Percentage of MDP (I), cMoP (J), GP (K) and Ly6C^hi^ monocytes (L) and frequency and total numbers of neutrophils (M) in the BM. (N-O) Frequency and total LKS cells (N), as well as the frequency and total cell number of the HSC/progenitor fractions in BCG versus *Mtb*-infected mice in the spleen after 28 days (O). Differences assessed by Two-tailed Student’s T-Test for each individual cell population in B-D and G-O or One-way ANOVA followed by Tukey’s Multiple Comparisons Test in F.

To quantify these potential effects, we directly compared the effect of *Mtb*-i.v. and BCG-i.v. on HSC and MPP kinetics (gating strategy in Figure S1A). *Mtb* or BCG in the BM correlated with significant expansion of the LKS population until at least 28 days post-infection (Figure 1H). LKS expansion was associated with proliferation, as shown by the increased frequency of Ki67^+^ LKS cells in *Mtb*-i.v. mice compared with BCG-i.v. and control mice on day 7 (Figure 1I) and was not due to an increase in long-term HSCs (LT-HSCs) but, rather, short-term HSCs (ST-HSCs) and MPPs (Figures S1B–S1D). Despite a more rapid response in *Mtb-*i.v. compared with BCG-i.v. mice on day 7 post-infection, by day 28, the numbers of HSCs and MPPs were similar in two groups (Figures S1B–S1D). BCG-i.v. protection against *Mtb* correlates with HSC skewing toward myelopoiesis by enhancing the pool of myeloid-biased MPP3s rather than lymphoid-biased MPP4s (Kaufmann et al., 2018). Unexpectedly, despite the greater virulence of *Mtb* compared with BCG, the dynamics of MPP3 versus MPP4 were indistinguishable between BCG-i.v. and *Mtb*-i.v. (Figures S1B–S1D). Thus, both bacteria access and persist equally in the BM to regulate HSC responses.

LKS cells give rise to all hematopoietic cells, including common myeloid progenitors (CMPs) and common lymphoid progenitors (CLPs), which lead to mature leukocytes. Because the plasticity between MPPs is high (Lai and Kondo, 2006; Pietras et al., 2014), lineage restriction is more stringently completed downstream at the CMP/CLP level (Akashi et al., 1997; Kondo et al., 1997). We next investigated how i.v. *Mtb* or BCG affected lineage-restricted progenitors. As with LKS cells (Figure 1D), neither *Mtb* nor BCG infected cKit^+^ myeloid progenitors (Figure S1E). Despite this, on day 7 and continuing on days 14 and 28 post-infection, *Mtb*, but not BCG, significantly suppressed myelopoiesis and promoted lymphopoiesis via loss of CMPs and granulocyte-monocyte progenitors (GMPs) and expansion of CLPs (Figures 1J–1L). Then we sought to investigate how *Mtb*, in contrast to BCG, caused these hematopoietic changes. RD1 is present in all virulent strains of *Mtb* but absent in BCG. Deletion of this region (H37Rv:ΔRD1) results in attenuation of *Mtb* that resembles BCG in cultured macrophages and mice (Lewis et al., 2003; Sherman et al., 2004). To see whether RD1 was responsible for the alterations in hematopoiesis, we infected mice with H37Rv:ΔRD1 and found no difference between *Mtb*-ΔRD1, *Mtb*, and BCG in expanding LKS or HSC populations 28 days post-infection (Figures 1M, 1N, and S1F–S1H). However, skewing of hematopoiesis by *Mtb* was completely dependent on RD1 expression because BCG and *Mtb-*ΔRD1 were indistinguishable (Figures 1N and S1G), whereas *Mtb* significantly suppressed myelopoiesis and promoted lymphopoiesis compared with *Mtb-*ΔRD1 (Figure S1H).

Downstream of CMPs/GMPs, granulocyte progenitors (GPs) give rise to neutrophils, whereas macrophage/dendritic cell progenitors (MDPs) and common monocyte progenitors (cMoPs) differentiate into all cells of the mononuclear phagocyte lineage or Ly6C^hi^ monocytes, respectively. Correlating with the reduction in CMPs/GMPs, on day 28 post*-Mtb* infection, we observed a significant reduction in the frequency and number of MDPs, cMoPs, and GPs and the number of mature Ly6C^hi^ monocytes and neutrophils (Figures 1O–1R and S1I–S1M). Therefore, loss of myeloid progenitors leads to loss of mature myeloid cells in the BM.

Under stress, the spleen can contribute to extramedullary hematopoiesis (Bronte and Pittet, 2013). To investigate whether the spleen may compensate for the suppressed BM myelopoiesis during systemic *Mtb* infection, we phenotyped splenic progenitors on day 28 after BCG-i.v. or *Mtb*-i.v. Following *Mtb* infection, we observed a smaller proportion of LKS cells in the spleen than in the BM that was equal between groups (Figure S1N) and, similar to BM, exhibited loss of CMPs/GMPs and expansion of CLPs (Figure S1O). Thus, *Mtb* appears to universally regulate hematopoiesis to restrict myelopoiesis.

### *Mtb* Uniquely Reprograms HSCs and Impairs Trained Macrophage Immunity

**Figure 2:**
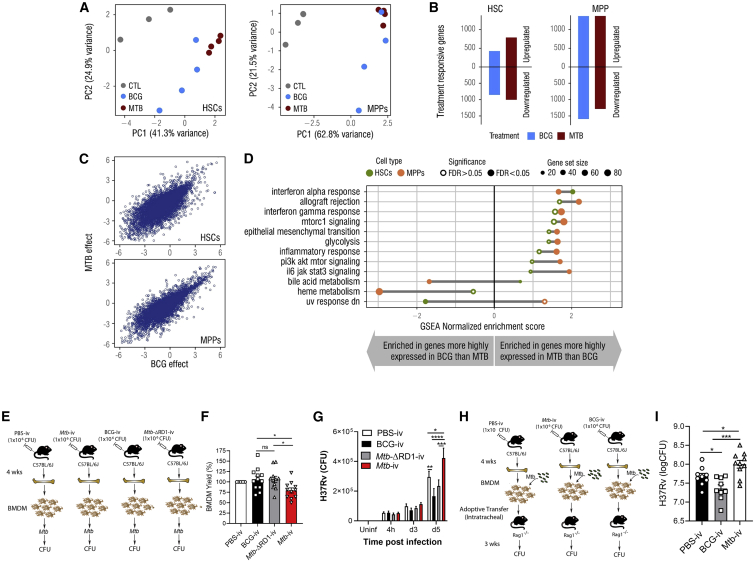
*Mtb*-i.v. Imprints a Unique Transcriptional Signature in HSCs and Detrimentally Trains BMDMs (A) Principal-component analysis (PCA) of gene expression of HSCs and MPPs from the BM of PBS, BCG-i.v., and *Mtb*-i.v. mice after 28 days. (B) Number of genes differentially up- or downregulated in HSCs and MPPs (FDR < 0.01). (C) Scatterplots of genome-wide effect sizes in HSCs (top) and MPPs (bottom). (D) GSEA of DEGs (FDR < 0.1). (E) BMDM model. (F) Relative BMDM yield. (G) CFUs from *in vitro Mtb*-infected BMDMs (n = 7–10 mice/group). (H) Adoptive transfer model. (I) Lung CFUs post-transfer. In (F) and (I), one-way ANOVA followed by Tukey’s multiple comparisons test was used. In (G), two-way ANOVA followed by Sidak’s multiple comparisons test was used. In (E)–(I), data are pooled from 2–3 independent experiment. See also Figure S2.

**Figure S2:**
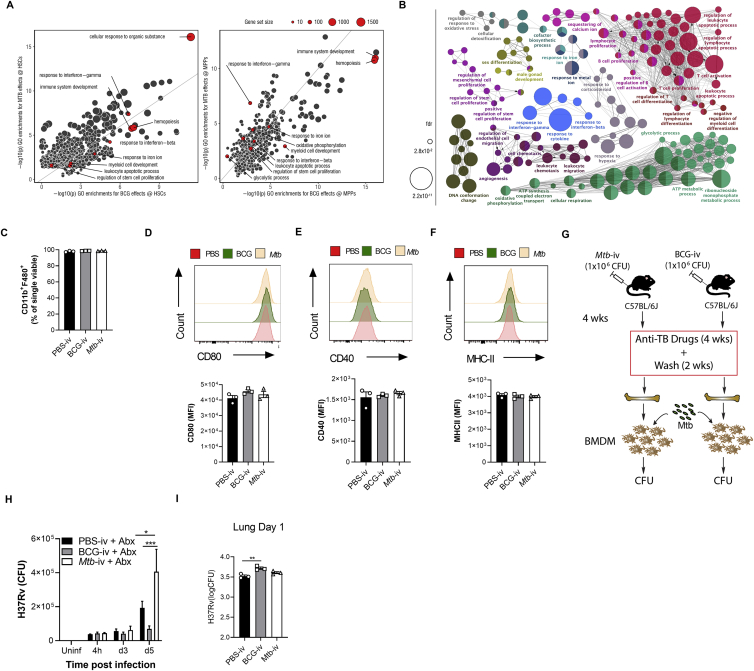
HSC Imprinting by BCG and *Mtb*-i.v. and Subsequent Anti-mycobacterial Responses by Macrophages to *Mtb* Infection *In Vitro*, Related to Figure 2 (A) Scatterplot for significance levels (-log10(p value) of gene ontology enrichment analyses conducted among DE genes upon BCG versus *Mtb* infections in HSC (left) and MPP (right). (B) Gene ontology terms enriched among DEG in response to *Mtb* in MPP (at FDR < 0.01). (C-F) BMDM from PBS control, BCG-iv and *Mtb*-iv groups were generated. Purity of BMDM cultures as determined by flow cytometry using expression of BMDM markers CD11b and F4/80 (C). Activation of mature BMDM was assessed by flow cytometry via MFIs of CD80 (D), CD40 (E) and MHC-II (F) with representative histograms in the top panels. Model of *in vivo* antibiotic treatment (G). BMDM-derived from the BM cells of these mice were infected with *Mtb* (H37Rv; MOI 1) and the number of CFU was determined at different time points after infection (H). (I) BMDM CFU prior to intratracheal transfer, as detailed in Figures 2H and 2I. Differences determined by One-way ANOVA followed by Tukey’s Multiple Comparisons Test in C-F and I; Two-way ANOVA followed by Tukey’s Multiple Comparisons Test in H.

Because of the similar responses at the cellular level in HSCs but divergence at the progenitor level, we postulated that *Mtb* transcriptionally reprogrammed HSCs to modulate downstream progenitors. To test this, we performed bulk RNA sequencing (RNA-seq) on HSCs (LKS^+^CD150^+^) and MPPs (LKS^+^CD150^−^) isolated on day 28 post- BCG-i.v., *Mtb*-i.v., or PBS-i.v. (control). For MPPs and HSCs, the first principal component (PC1) of gene expression data explained over 40% of the total variance and segregated *Mtb*-i.v. versus BCG-i.v. or PBS control mice (Figure 2A; one-tailed t test, p = 0.023 and 5.1 × 10^−7^ for HSCs and MPPS, respectively). Accordingly, we found extensive differences in gene expression levels between *Mtb*-i.v. and BCG-i.v. compared with PBS control mice (Figure 2B; *Mtb*-i.v. versus control: 1,282 [10.6%] and 2,952 [24.3%] differentially expressed genes (DEGs) in HSCs and MPPs, respectively; BCG-i.v. versus control: 1,793 [14.8%] and 2,646 [21.8%] DEGs in HSCs and MPPs, respectively; Table S1; false discovery rate [FDR] < 0.01). Regulation of gene expression by *Mtb* and BCG was highly concordant in MPPs and HSCs (Figure 2C; Pearson correlation of *Mtb*-i.v. versus BCG-i.v. effects: 0.751 and 0.837 in HSCs and MPPs, respectively, p < 1 × 10^−10^ in both) as well as the Gene Ontology terms enriched among genes that significantly changed expression levels in response to *Mtb* or BCG (Figure S2A; Pearson correlation of significance levels: r = 0.68 [HSCs], r = 0.77 [MPPs], p < 1 × 10^−10^). Globally, DEGs in BCG-i.v. or *Mtb*-i.v. infection were strongly enriched for gene sets involved in IFN-I and IFN-II responses, T cell activation, proliferation, and glycolysis (Figure S2B; Table S2), suggesting that the presence of mycobacteria in the BM led to IFN-dependent rewiring of HSCs and MPPs, consistent with our previous report (Kaufmann et al., 2018).

Despite the overall similarities in the gene expression response of HSCs and MPPs to BCG and *Mtb*, we found significant differences in genes that respond to *Mtb*-i.v. or BCG-i.v. 689 of these infection-responsive genes in HSCs and 402 in MPPs showed significant differences in expression across mycobacteria (FDR < 10%; Table S1). Gene set enrichment analyses (GSEAs) showed that genes involved in IFN-I signaling, glycolysis, and inflammation were significantly enriched among genes more highly expressed in response to *Mtb*-i.v. compared with BCG-i.v. (Figure 2D; Table S3). In contrast, DEGs following BCG-i.v. versus *Mtb*-i.v. were enriched for those involved in heme metabolism (Figure 2D). These data, suggested that intrinsic differences in the transcriptional magnitude of pathways involved in IFN-I signaling and heme in HSC/MPP populations might be transmitted to the more committed progenitors and effector cells, modulating their numbers and/or function.

To test the potential contribution of these transcriptional changes in HSCs on the functional capacity of innate immunity, we next generated bone marrow-derived macrophages (BMDMs) from BCG-i.v., *Mtb-*ΔRD1, *Mtb*-i.v., and PBS mice and infected them with *Mtb* (Figure 2E). To ensure that there was no contamination with mycobacteria from the *in vivo* infection, we cultured cells with anti-TB drugs, and all BMDM cultures were *Mtb* or BCG free at the time of *in vitro* infection (Figure 2G). Similar to the results of *in vivo* myelopoiesis (Figures 1J, 1K, and 1O–1R), *Mtb-*i.v. mice generated fewer BMDMs than BCG-i.v. or *Mtb-*ΔRD1-i.v. mice relative to control mice (Figure 2F). Moreover, despite culturing the same number of BMDMs in each group prior to *in vitro Mtb* infection, BMDMs from *Mtb-*i.v. mice failed to control *Mtb* growth on day 5 post-infection in an RD1-dependent manner (Figure 2G). As expected, BMDMs derived from BCG-i.v. mice showed enhanced protection against *Mtb* infection. Although we observed a decrease in the number of BMDMs generated in the *Mtb*-i.v. group (Figure 2F), the purity and activation levels (CD80, CD40, and major histocompatibility complex [MHC] class II) were similar (Figures S2C–S2F). Therefore, the impaired training of BMDMs in the *Mtb*-i.v. group is not due to the presence of immature cells or contamination by other cell types. To investigate whether this phenomenon required constant exposure to the bacteria *in vivo*, we treated BCG-i.v., *Mtb*-i.v., and control mice with anti-TB drugs for 28 days, followed by 2 weeks of rest. BM and BMDM cultures were free of mycobacteria (*Mtb* or BCG). BMDMs from these mice were then infected with *Mtb in vitro* (Figure S2G), and again, BMDMs from *Mtb*-i.v. mice showed impaired capacity to control *Mtb* growth (Figure S2H).

Finally, to assess the functional anti-*Mtb* capacity of BMDMs *in vivo*, we performed adoptive transfers. We differentiated BMDMs in the presence of anti-TB drugs from the PBS, BCG-i.v., and *Mtb*-i.v. groups on day 28, infected the BMDMs *in vitro* with *Mtb*, and transferred them (intratracheally) into RAG1-deficient mice (lacking B and T cells) (Figure 2H). As observed previously (Kaufmann et al., 2018), BMDMs from the BCG-i.v. group provided enhanced protection after 21 days (Figure 2I). However, mice that received BMDMs from the *Mtb*-i.v. group had significantly higher bacterial burdens in the lungs than PBS and BCG-i.v. mice. This difference was observed despite the number of *Mtb* in macrophages from the BCG-i.v. group being slightly higher than in the PBS or *Mtb*-i.v. group prior to transfer (Figure S2I). Thus, *Mtb*, in contrast to BCG, suppresses myelopoiesis to impair the generation and intrinsic antimycobacterial capacity of BMDMs *in vitro* and *in vivo*, uncovering a novel strategy of *Mtb* pathogenesis in the BM.

### Pulmonary *Mtb* Infection Suppresses Myelopoiesis and Prevents Trained Immunity

**Figure 3:**
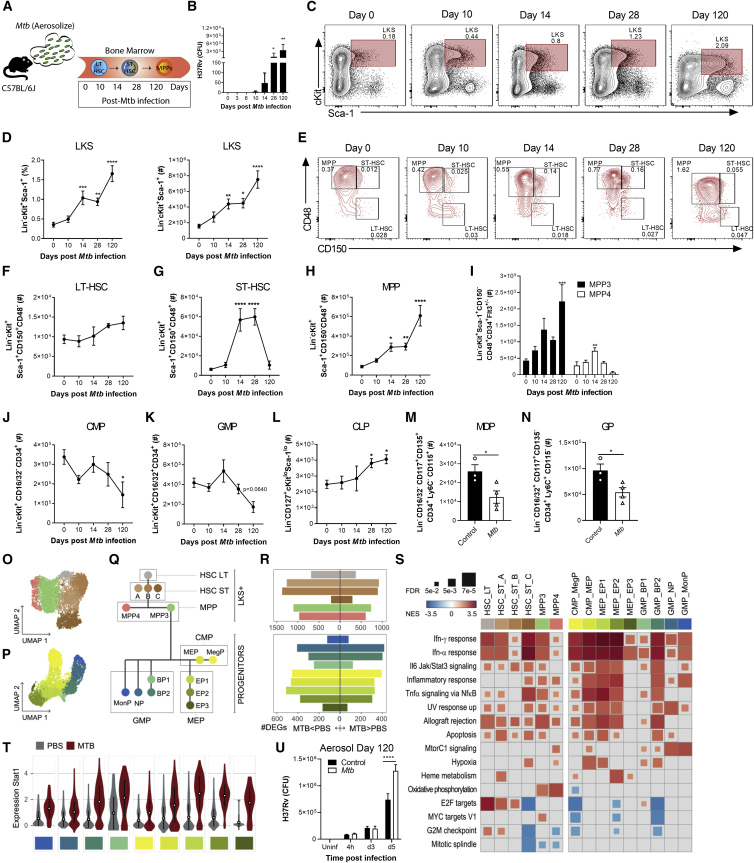
Aerosolized *Mtb* Disseminates to the BM and Expands LKS Cells (A) Aerosol model used in (B)–(U). (B) BM CFUs. (C and D) Representative FACS plots of LKS cells as quantified in (D) as frequency (left) and total number (right). (E–I) Representative FACS plots (E) of LT-HSCs (F), ST-HSCs (G), MPPs (H), and MPP3/4 (I) (n = 4–10 mice/group). (J–N) Total CMPs (J), GMPs (K), and CLPs (L) (n = 4–8 mice/group) and MDPs (M) and GPs (N) on day 120. (O and P) UMAP dimensionality reduction plots for LKS^+^ (O) and cKit^+^ (P) cells. (Q) Hematopoietic tree with approximate position of the different clusters identified (color code in common with O and P). (R) Number of genes up- and downregulated in each cluster (abs(logFC) > 0.1 and FDR < 0.05). (S) GSEA of genes ranked according to *Mtb* effects in each cluster. (T) Stat1 expression across clusters. (U) BMDMs from mice infected with *Mtb* for 120 days were infected *in vitro* and CFU quantified (n = 3 mice/group). One-way ANOVA followed by Tukey’s multiple comparisons test (B, D, and F–L), two-tailed Student’s t test (M and N), and two-way ANOVA followed by Sidak’s multiple comparisons test (U) were used. Data are representative of two (B and J–N) or three (C–I) independent experiments. See also Figure S3.

**Figure S3:**
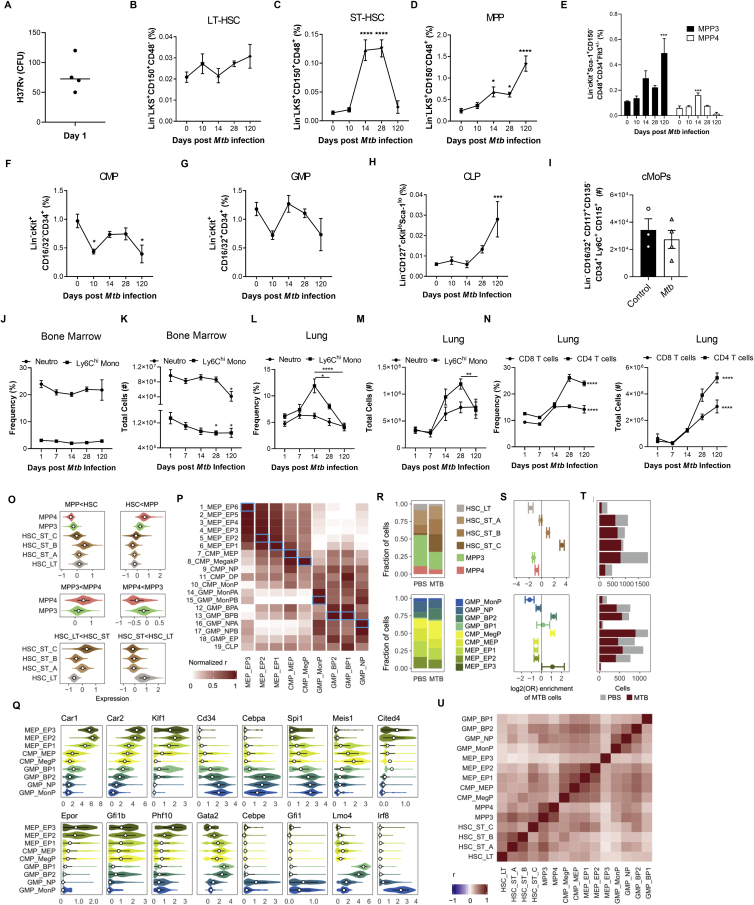
HSC and Progenitor Kinetics following Aerosolized *Mtb* Infection, Related to Figure 3 (A-M) WT mice were infected with aerosolized *Mtb.* (A) Day 1 lung CFU following aerosol infection. (B-H) Kinetics of the frequencies among BM cells of LT-HSC (B), ST-HSC (C), MPP (D), MPP3/MPP4 (E), CMP (F), GMP (G), CLP (H) in *Mtb* infected WT mice. (I) Total cell counts of cMoPs in the BM of WT mice at 120 days post-infection. Kinetics of the frequencies and total numbers of neutrophils and Ly6C^hi^ monocytes in the BM (J-K) and lung (L-M) as well as the frequencies and total cell counts of adaptive CD8 and CD4 T cells in the lung (N). (O) Average expression of cell-type markers across clusters. (P) Correlations between genome-wide expression patterns of our myeloid progenitor data and results published by Paul et al. (2015). In each column, Spearman correlations are normalized to cover the range [0-1]. Blue boxes mark the best fit (i.e., candidate identity match) for each of our clusters. (Q) Expression patterns across clusters for some marker genes associated to commitment to the different lineages characterized in this study. (R) Proportion of cells per cluster in both sub-populations (LKS and myeloid progenitors), for cells coming from PBS versus *Mtb* treated mice. (S) Fisher exact test enrichments (log2 odds ratios) for the fraction of *Mtb* cells in each cluster. (T) Number of PBS versus *Mtb* cells in each cluster. (U) Genome wide correlation of *Mtb* effect sizes (logFC) across clusters. Statistics were (B-H, J-N) One-way ANOVA followed by Tukey’s Multiple Comparisons Test with significance shown compared to day 0 or day 1 post-infection mice for each cell type and Two-tailed Student’s t test (I).

The systemic model of *Mtb* infection does not represent the natural route of infection in humans. To extend our findings to a physiological model of pulmonary TB, we infected mice with a low dose of *Mtb* via the aerosol route (Figures 3A and S3A). Within 10–14 days following aerosolized *Mtb* infection, bacteria disseminate to the lung-draining lymph nodes by infected dendritic cells and/or monocytes, leading to T cell priming (Chackerian et al., 2002; Samstein et al., 2013; Wolf et al., 2008). Interestingly, we found that *Mtb* also reached the BM 10 days post-infection and grew until the last time point analyzed at 120 days, when the number of *Mtb* in the BM was close to the i.v. model (Figure 3B). As with the i.v. model, this was associated with expansion of LKS cells (Figures 3C and 3D), ST-HSCs, and MPPs but not LT-HSCs (Figures 3E–3H and S3B–S3D). Moreover, aerosolized *Mtb* skewed hematopoiesis toward MPP3 (Figure 3I and S3E). In the lineage-restricted progenitors, we again observed a reduction of CMPs/GMPs and expansion of CLPs (Figures 3J–3L and S3F–S3H) as well as a significant loss in the numbers of MDPs and GPs (Figures 3M and 3N) and a trending decrease in cMoPs (Figure S3I). This reduction of myelopoiesis led to a decreased number of mature neutrophils and Ly6C^hi^ monocytes in the BM on day 120 compared with uninfected cells (Figures S3J and S3K) as well as loss of Ly6C^hi^ monocytes from peak levels (day 14 versus 120) in the lungs, whereas T cells increased continually (Figures S3L–S3N). Thus, *Mtb* enters the BM and suppresses myelopoiesis regardless of infection route, and hematopoietic biasing may contribute to driving the shift in the pulmonary immune response from innate to adaptive immunity.

Next we sought to characterize, at single-cell resolution, the transcriptional response of HSCs and progenitor cells at 120 days of *Mtb* aerosol infection. To do so, we performed droplet-based single-cell RNA-seq (scRNA-seq) (Zheng et al., 2017) on sorted BM LKS cells and cKit^+^ progenitors. Following quality control, we kept a total of 5,698 high-quality LKS cell transcriptomes (LKS dataset: 2,388 derived from control and 3,310 derived from the *Mtb* group) and 5,745 transcriptomes from cKit^+^ cells (progenitor dataset: 2,241 PBS and 3,504 *Mtb*). LKS cells were classified into six clusters based on known canonical markers as well as DEGs between subsets of LKS cells (Cabezas-Wallscheid et al., 2014; STAR Methods; Figure S3O): a cluster of long-term HSCs (HSC-LT), three clusters of short-term HSCs (HSC-ST_A, B, and C), one cluster of lymphoid-biased MPP4, and one cluster of myeloid-biased MPP3 (Figures 3O and 3Q). Among cKit^+^ cells, we identified two clusters of CMPs, four clusters of GMPs, and three clusters of megakaryocyte-erythrocyte progenitors (MEPs) (Figures 3P, 3Q, S3P, and S3Q). In accordance with the flow cytometry data, we observed an expansion of ST-HSCs (up to log2(OR) = 3.3 in HSC-ST_C, p < 2.2E−16) and reduction of monocyte precursors (GMP_MonPs, log2(OR) = −1.2, p < 1E−7) in the *Mtb* group compared with PBS (Figures S3R and S3S).

The gene expression across all clusters was markedly changed between cells derived from *Mtb* infected and control mice (Table S4). LKS cells were the most responsive to *Mtb* infection (Figure 3R, top panel), showing between 506 (HSC-ST_A) and 2,269 DEGs (HSC-ST_B) (FDR < 5% and absolute fold change ≥ 0.1). Aerosolized *Mtb* also largely affected the transcriptional profile of cKit^+^ cells, ranging from 203 DEGs among MonPs (cluster GMP_MonP) and 851 DEGs among CMP_MegP (Figure 3R, bottom panel).

Despite differences in the number of DEGs identified within each cell type—which, in part, reflects differences in statistical power because of differences in cell numbers (Figure S3T)—the response to aerosolized *Mtb* was concordant across all cell types, with stronger correlations among cell types that are functionally more related. For example, the correlation in the transcriptional response to *Mtb* between MPP3 and MPP4 cells was 0.76 (p < 2.2E−16) compared with only 0.48 (p < 2.2E−16) between MPP3 and LT-HSCs (Figure S3U). GSEA revealed that the two pathways most enriched among genes upregulated in response to aerosolized *Mtb* were the IFN-II and IFN-I pathways (enriched in all clusters; normalized enrichment score [NES] across clusters ranges from 1.51–3.23, FDR < 0.05) (Figure 3S). *Stat1*, a key TF involved in the response to IFN, was significantly upregulated in response to *Mtb* in virtually all clusters (Figure 3T), further highlighting the importance of IFN signaling in reprograming HSCs and progenitors. As with *Mtb*-i.v. infection, this imprinting in the BM correlated with loss of *in vitro* control of *Mtb* by BMDMs from aerosol *Mtb*-infected mice (Figure 3U). Aerosol *Mtb* also imprints HSCs/progenitors to blunt myelopoiesis and generate macrophages that fail to control *Mtb* growth.

### IFN-I Signaling Restricts Myelopoiesis and Impairs Macrophage Responses to Confer Susceptibility to *Mtb* Infection

**Figure 4:**
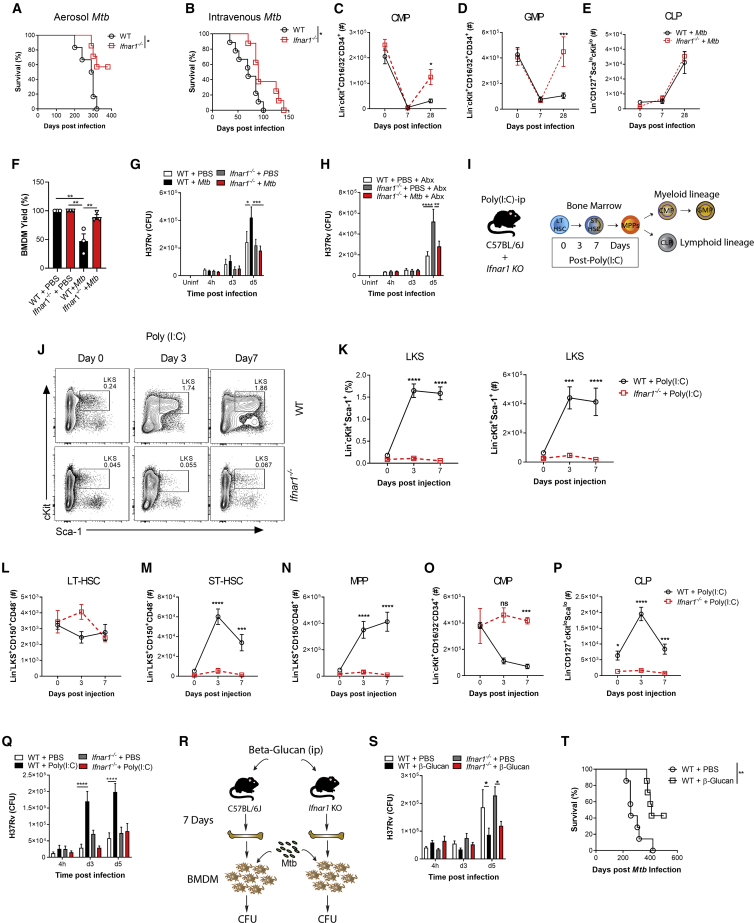
*Mtb* Suppresses Myelopoiesis and Impairs Innate Training in an IFN-I-Dependent Manner (A and B) Survival following (A) aerosol (n = 6–7 mice/group) or (B) i.v. (n = 8–9 mice/group) infection. (C–E) Total CMPs (C), GMPs (D), and CLPs (E) after i.v. infection. (F) Relative BMDM yield. (G) *In vitro* BMDM CFUs with Abx in culture. (H) *In vitro* BMDM CFUs with Abx *in vivo*. (I) Poly(I:C) model. (J and K) Representative FACS plots of LKS cells as quantified in (K) with percentages (left) and totals (right). (L–P) Total LT-HSCs (L), ST-HSCs (M), MPPs (N), CMPs (O), and CLPs (P). (Q) *In vitro* BMDM CFUs. (R) β-Glucan model. (S) *In vitro* BMDM CFUs (n = 6–7 replicates/group). (T) Aerosol survival (7 mice/group). In (A), (B), and (T), log rank test was used. In (C)–(E), (G), (H), (K)–(Q), and (S), two-way ANOVA followed by Sidak’s multiple comparisons test was used. In (F), one-way ANOVA followed by Tukey’s multiple comparisons test was used. Data are representative of two (S) or three (C–Q) independent experiments. See also Figure S4.

**Figure S4:**
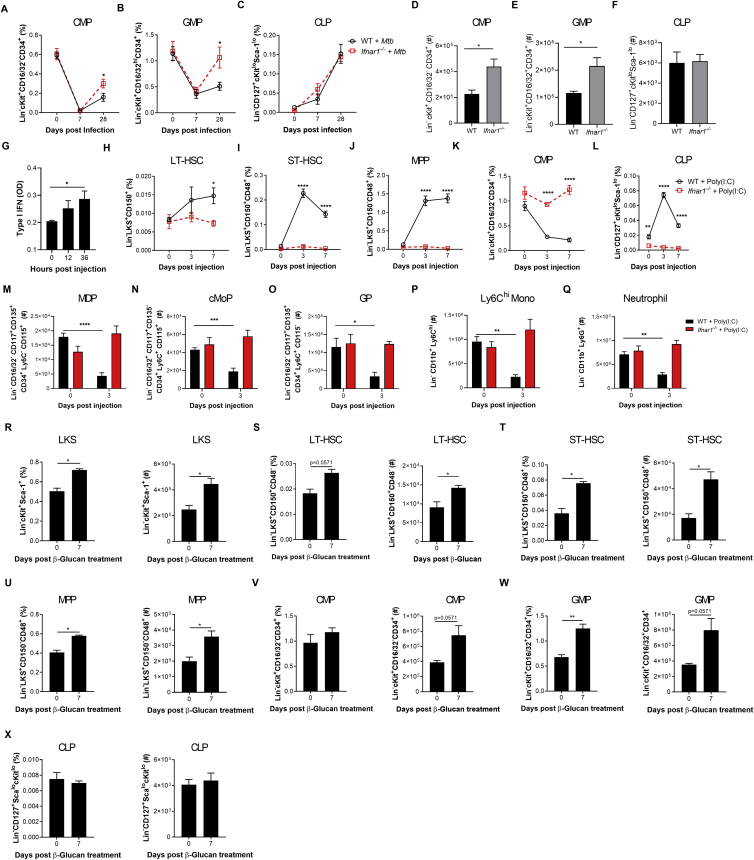
IFN-I Signaling Impairs Myelopoiesis, Related to Figure 4 (A-C) WT and *Ifnar1*^*−/−*^ mice were infected with 1x10^6^*Mtb*-iv. Kinetics of the frequencies among single viable BM cells of CMP (A), GMP (B) and CLP (C). (D-F) mice were infected with *Mtb* via the aerosol route. Percentages of CMP (D), GMP (E) and CLP (F) in the BM at day 60. (G) Active IFN-I was measured in the BM collected after 12, 24, and 36 hours after Poly (I:C) treatment. (H-Q) WT and *Ifnar1*^−/−^ mice were treated with Poly (I:C), or PBS, at day 0, 2, 4, 6. Frequencies among single viable BM cells of LT-HSC (H), ST-HSC (I), MPP (J), CMP (K) and CLP (L) at days 0, 3 and 7, as well as the total number of MDP (M), cMoP (N), GP (O), Ly6C^hi^ monocytes (P) and neutrophils (Q) at day 3 post-Poly-(I:C) treatment. (R-X) WT mice were treated with β-glucan at day 0 and day 3. Frequencies among BM cells (left panel) and total cell counts (right panel) of LKS (R), LT-HSC (S), ST-HSC (T), MPP (U), CMP (V), GMP (W) and CLP (X) in PBS (day 0) or β-glucan i.p. at day 7 post-treatment. Differences determined by Two-way ANOVA followed by Sidak’s Multiple Comparisons Test in A-C, H-Q, Two-Tailed Student’s T-Test in D-F, One-way ANOVA followed by Dunnett’s Multiple Comparisons Test in G and Non-Parametric Mann-Whitney Test R-X.

The RNA-seq analyses indicated that *Mtb* significantly modulated IFN. IFN-II is critical for HSC expansion and myelopoiesis (Baldridge et al., 2010; Belyaev et al., 2010; Kaufmann et al., 2018), whereas IFN-I induces loss of stem cell numbers and function (Pietras et al., 2014; Smith et al., 2018). Interestingly, during *Mtb* infection, IFN-II signaling is essential for host survival (Nandi and Behar, 2011), whereas IFN-I signaling is detrimental (Antonelli et al., 2010). Thus, we wondered whether *Mtb*-induced IFN-I directly affected lineage commitment and BMDM anti-microbial responses. Following aerosol or i.v. *Mtb* infection, *Ifnar1*^*−/−*^ mice exhibited enhanced survival (Figures 4A and 4B) and a significant increase in CMPs and GMPs but no difference in CLPs compared with the WT (Figures 4C–4E and S4A–S4F). Thus, additional factors other than IFN-I promote lymphopoiesis during *Mtb* infection.

It has been shown that IFN-I signaling impairs macrophage anti-*Mtb* immunity (Mayer-Barber et al., 2014) and also enhances susceptibility to *Mtb* infection via recruitment of permissive BM-derived CCR2^+^ monocytes into the lungs (Antonelli et al., 2010). Thus, we investigated how IFN-I signaling may regulate BMDM trained immunity. *Mtb*-i.v. infected *Ifnar1*^*−/−*^ mice had an enhanced *in vitro* BMDM yield compared with WT *Mtb*-i.v. mice (Figure 4F), and, using either our *ex vivo* (Figure 2E) or *in vivo* model (Figure S2G), the lack of protection by BMDMs from *Mtb*-infected mice was entirely dependent on IFN-I signaling (Figures 4G and 4H).

To determine the specific role of IFN-I in hematopoiesis, we used a model of systemic IFN-I production through the intraperitoneal administration of the TLR3 synthetic viral ligand polyinosinic:polycytidylic acid (poly(I:C)). The effects on hematopoiesis were then assessed on days 3 and 7 post-poly(I:C) treatment (Figure 4I; Smith et al., 2018). In line with previous reports (Pietras et al., 2014), an increase in BM IFN-I was observed in mice shortly following poly(I:C) administration (Figure S4G). Similar to *Mtb*, poly(I:C) increased the LKS/ST-HSCs/MPPs but not LT-HSCs (Figures 4J–4N and S4H–S4J) and decreased CMPs while increasing CLPs (Figures 4O, 4P, S4K, and S4L). Poly(I:C)-treated WT mice also displayed reduced MDPs, cMoPs, and GPs as well as Ly6C^hi^ monocytes and neutrophils (Figures S4M–S4Q). As expected, all effects were entirely IFN-I dependent, as revealed in *Ifnar1*^*−/−*^ mice (Figures 4J–4P and S4H–S4Q). As with *Mtb*-infected mice, BMDMs from poly(I:C)-treated WT mice showed IFN-I-dependent impaired resistance to *Mtb* (Figure 4Q). Thus, IFN-I signaling inhibits myelopoiesis and prevents generation of protective trained immunity.

To contrast the effect of poly(I:C) on suppression of the myeloid lineage and trained immunity, we next used β-Ggucan (Figure 4R), which has been shown to promote myelopoiesis (Mitroulis et al., 2018; Moorlag et al., 2020). β-Glucan-treated mice showed enhanced LKS cells/LT-HSCs/ST-HSCs/MPPs 7 days post-treatment (Figures S4R–S4U) and myeloid CMPs/GMPs (Figures S4V and S4W) with no changes in CLPs (Figure S4X). We generated BMDMs from β-glucan-treated or PBS mice and infected them with *Mtb in vitro*. BMDMs from β-glucan-treated mice protected against *Mtb* infection via an IFN-I-independent (Figure 4S) but IL-1-depedent mechanism (Moorlag et al., 2020). Remarkably, the effect of β-glucan was long lived *in vivo* with aerosol *Mtb* infection; 60% of β-glucan-treated mice survived up to 500 days (Figure 4T). There are multiple signaling pathways involved in hematopoietic stem and progenitor cell imprinting to promote myelopoiesis and generate protective trained immunity (e.g., IFN-II or IL-1) or suppress myelopoiesis and generate impaired trained immunity (e.g., IFN-I) against *Mtb*.

### IFN-I Signaling Modulates Iron Metabolism to Limit Myelopoiesis and Enhance Susceptibility to *Mtb*

**Figure 5:**
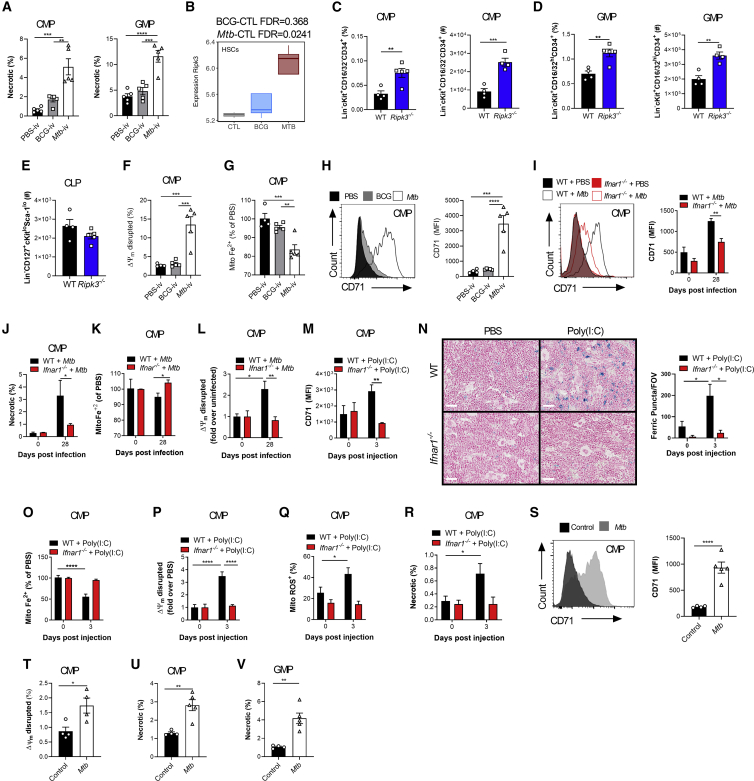
IFN-I Regulates Fe Metabolism in Myeloid Progenitors and Triggers Necrosis (A) Frequencies of necrotic (NucSpot^+^ AnnexinV^−^) CMPs (left) and GMPs (right) on day 7. (B) *Ripk3* expression in HSCs. (C–H) Mice were infected i.v. for 7 days. (C–E) Percentage (left) and total (right) of CMPs (C) and GMPs (D) and total CLPs (E). (F) CMPs with disrupted MMP. (G) Relative mitochondrial Fe^2+^ in CMPs. (H) Representative histograms (left) and quantification (right) of CMP CD71 expression. (I–L) WT and *Ifnar1*^*−/−*^ mice were infected i.v. for 28 days. (I) Representative histograms (left) and quantification (right) of CMP CD71 expression. (J) Percentage of necrotic CMPs. (K) Relative mitochondrial Fe^2+^ in CMPs. (L) CMPs with disrupted MMP. (M–R) WT and *Ifnar1*^*−/−*^ mice were treated with poly(I:C) for 3 days. (M) CMP CD71 expression. (N) Representative micrographs (left) and total puncta (right) of BM Perl’s Prussian Blue; the scale bar represents 50 μM. (O) Relative mitochondrial Fe^2+^ in CMPs. (P) CMPs with disrupted MMP. (Q and R) Frequency of ROS^+^ (Q) or necrotic (R) CMPs. (S–V) WT mice were infected with aerosolized *Mtb* for 120 days. (S) Representative histograms (left) and quantification (right) of CMP CD71 expression. (T) CMPs with disrupted MMP. (U and V) Percentage of necrotic CMPs (U) or GMPs (V). In (A) and (F)–(H), one-Way ANOVA followed by Tukey’s comparison test was used. In (C)–(E) and (S)–(V), two-tailed Student’s t test was used. In (I)–(R), two-way ANOVA followed by Sidak’s, Tukey’s, or Dunnett’s multiple comparisons test was used. Data are representative of two (A and F–I) independent experiments or are pooled from 2–3 individual experiments (M–R). See also Figure S5.

**Figure S5:**
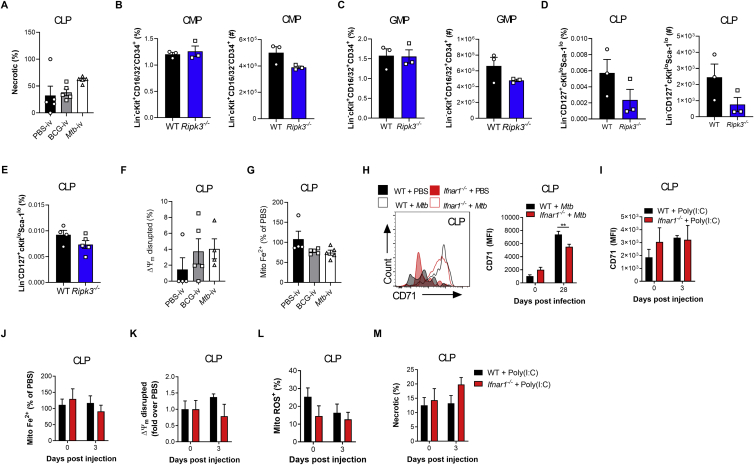
The IFN-I/Iron Axis Regulates Cell Death of Myeloid Progenitors, Related to Figure 5 Frequency of necrotic (NucSpot+ AnnexinV^-^) CLP (A) in the BM of BCG-iv and *Mtb*-iv infected mice at day 7. (B-D) Frequency among single viable BM cells (left panels) and total cell counts (right panels) of CMP (B), GMP (C), CLP (D) of naive WT and *Ripk3*^−/−^ mice. (E) Frequency among BM cells of CLP in WT and *Ripk3*^−/−^*Mtb*-iv infected mice at day 7. (F) Frequency of CLP with disrupted mitochondria in the BM of BCG-iv and *Mtb*-iv infected mice at day 7. (G) Relative levels of mitochondrial iron (Fe^2+^) in CLP of BCG-iv and *Mtb*-iv infected mice at day 7. (H) Representative histogram of expression of CD71 (left panel) and quantification of CD71 expression (right panel) on CLPs in the BM of *Mtb*-iv infected WT and *Ifnar1*^−/−^ mice at day 28. (I) Expression of CD71 on CLPs in the BM of WT and Poly (I:C)-treated WT and *Ifnar1*^−/−^ mice. (J) Relative proportion of mitochondrial iron dye (Fe^2+^) in the CLP of Poly (I:C)-treated WT and *Ifnar1*^−/−^ mice. Frequency of CLPs with a disrupted mitochondrial potential (K), high mitochondrial ROS (L), or those that are necrotic (M). Differences were assessed via One-way ANOVA followed by Tukey’s Multiple Comparison Test (A; F-G), Two-tailed Student’s T-Test (B-E), Two-way ANOVA followed by Sidak’s Multiple Comparisons Test (H-M).

IFN-I signaling has been shown to regulate progenitor cell death during bacterial infection (Smith et al., 2018), and cell death programs are critical in HSC lineage decisions (Kanayama et al., 2017). Thus, we investigated whether cell death limited myelopoiesis. Necrosis was significantly increased in CMPs/GMPs of *Mtb-*i.v. infected compared with BCG-i.v. mice (Figure 5A) but not in CLPs (Figure S5A). Interestingly, the expression levels of *Ripk3*, a key mediator of necroptosis (Vandenabeele et al., 2010), were increased significantly in HSCs from *Mtb*-i.v. but not BCG-i.v. mice (Figure 5B), which led us to speculate that the observed necrosis of CMPs/GMPs was RIPK3 dependent, as suggested by previous reports (Smith et al., 2018; Yamashita and Passegue, 2019). The numbers of CMPs/GMPs/CLPs in naive WT and *Ripk3*^*−/−*^ mice were similar (Figures S5B–S5D). However, 7 days post-*Mtb* infection, *Ripk3*^*−/−*^ mice had significantly increased frequencies and numbers of CMPs and GMPs compared with WT mice, with no effect on CLPs (Figures 5C–5E and S5E). Thus, *Mtb* induces RIPK3-mediated necroptosis in myeloid progenitors.

Our HSC RNA-seq revealed an enrichment for heme metabolism in BCG-i.v. compared with *Mtb-*i.v. mice (Figure 2D). Interestingly, dysregulation of Fe/heme metabolism in HSCs leads to oxidative stress through free radical generation (e.g., H_2_O_2_) and HSC ablation (Muto et al., 2017), whereas reduction in the cellular iron pool causes mitochondrial dysfunction and myeloid cell death (al-Rafaie et al., 1994; Martin-Sanchez et al., 2017). Thus, we postulated that *Mtb* disrupts Fe metabolism to limit myelopoiesis. We first found that necroptosis was accompanied by mitochondrial dysregulation in CMPs (Figure 5F) but not in CLPs (Figure S5F). This correlated with a reduction in mitochondrial Fe content in CMPs of *Mtb-*i.v. versus BCG-i.v. mice (Figure 5G) but not in CLPs (Figure S5G) and a concordant upregulation of the transferrin receptor (CD71), which plays a critical role in Fe import (Ganz, 2013; Figure 5H). This was at least partly IFN-I dependent because the levels of CD71 expression in CMPs and CLPs (Figures 5I and S5H) as well as the frequency of necrotic CMPs were also reduced significantly in *Mtb*-infected *Ifnar1*^*−/−*^ mice (Figure 5J). Additionally, the mitochondrial Fe level (Figure 5K) and mitochondrial membrane potential (MMP) (Figure 5L) remained equal to uninfected *Ifnar1*^−/−^ mice, confirming the importance of IFN-I signaling in CMP Fe metabolism. Similarly, the expression levels of CD71 were increased significantly in CMPs but not CLPs after poly(I:C) treatment in WT mice (Figures 5M and S5I). This was associated with Fe accumulation in the BM (Figure 5N) along with a reduction in mitochondrial Fe (Figures 5O and S5J) and MMP (Figures 5P and S5K) in CMPs, but not CLPs, in an IFN-I-dependent fashion. Loss of mitochondrial potential was furthermore linked to accumulation of mitochondrial reactive oxygen species (ROS) and necrosis in CMPs but not CLPs (Figures 5Q, 5R, S5L, and S5M). Similar results were obtained following aerosol infection (Figures 5S–5V).

**Figure 6:**
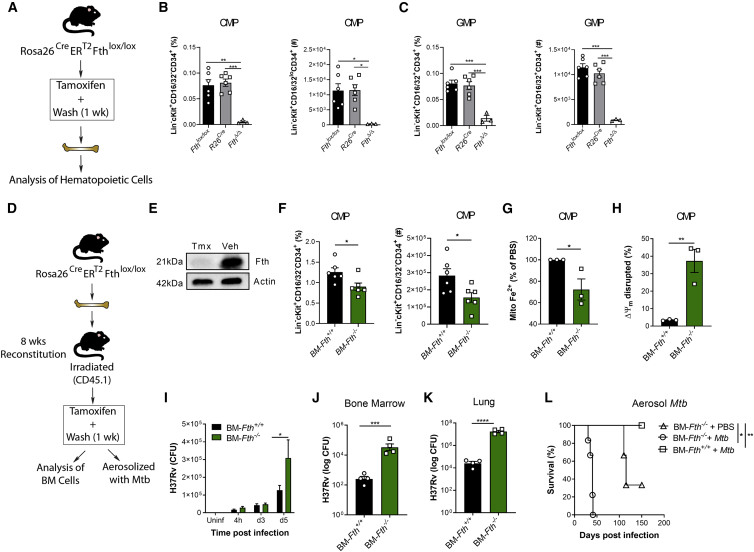
Iron Dysregulation in the BM Promotes Susceptibility to TB (A) *Fth*^*Δ/Δ*^ model. (B and C) Percentages (left) and numbers (right) of CMPs (B) or GMPs (C). (D) BM-*Fth*^−/−^ model. (E) BM ferritin immunoblot; actin = loading control. (F) Percentage (left) and total (right) of CMPs. (G and H) Relative mitochondrial Fe^2+^ (G) or disrupted MMP (H) in CMPs. (I) *In vitro* BMDM CFUs. (J and K) BM-*Fth*^+/+^ or BM-*Fth*^−/−^ mice were infected with aerosolized *Mtb* for 28 days. Shown are BM (J) and lung (K) CFUs. (L) Aerosol survival (n = 5 mice/group). One-way ANOVA followed by Tukey’s multiple comparisons test was used in (B) and (C). Two-tailed Student’s t test was used in (F)–(H), (J), and (K). Two-way ANOVA followed by Sidak’s multiple comparisons test (I) or log rank test (L) was used. Data are pooled from two experiments (B and C) or representative of two independent experiments (E, F–H, and I). See also Figure S6.

**Figure S6:**
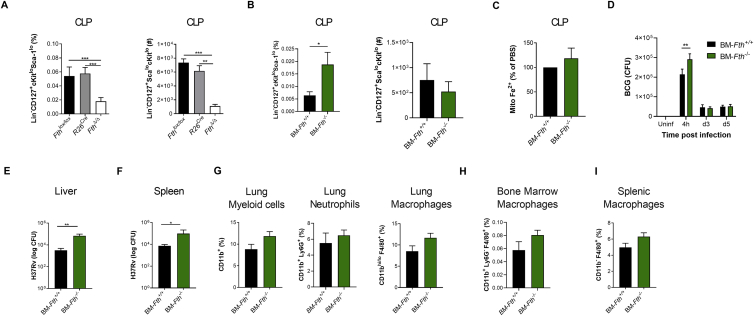
Loss of Iron Homeostasis Disrupts Myelopoiesis, Related to Figure 6 (A) Frequency (left panel) and total cell count (right panel) of CLP in *Fth*^Δ/Δ^, *R26*^cre^ or *Fth*^lox/lox^ mice as generated in Figure 6A. (B-G) BM-*Fth*^*+/+*^ or BM-*Fth*^−/−^ chimeric mice were generated as described in Figure 6D. Frequency (left panel) and total cell count (right panel) of CLP in BM-*Fth*^+/+^ or BM-*Fth*^−/−^ chimeric mice (B). (C) Relative percentage of iron (Fe^2+^) specific dye in the mitochondria of CLP in the BM of BM-Fth^−/−^ compared to BM-*Fth*^+/+^ mice. (D) BCG CFU (infection at MOI 10) in BMDM from BM-*Fth*^+/+^ or BM-*Fth*^−/−^ mice at the indicated time points. CFU in the liver (E) and spleen (F) following 28 days of aerosol *Mtb* infection. Frequency of myeloid cell populations in the lung (G), BM (H) and spleen (I) of naive BM-*Fth*^+/+^ and BM-*Fth*^−/−^ mice. Differences assessed by One-way ANOVA followed by Tukey’s Multiple Comparisons Test (A), Two-tailed Student’s T-Test (B-C and E-I), or Two-way ANOVA followed by Sidak’s Multiple Comparisons Test (D).

Having established that IFN-I signaling modulated Fe metabolism specifically within myeloid progenitors to induce necrosis, we next investigated whether disruption of Fe metabolism, using an inducible mouse model of ferritin H chain (FTH) deficiency (Blankenhaus et al., 2019), dysregulated hematopoiesis and/or trained immunity after *Mtb* infection. Whole-body tamoxifen-inducible *Fth* deletion via Cre recombinase activity under the ROSA26 promoter in adult mice (referred to as *Fth*^*Δ/Δ*^ mice) (Figure 6A) is lethal shortly after tamoxifen administration (Blankenhaus et al., 2019). Although the cause of death in *Fth*^*Δ/Δ*^ mice has been shown to be due to altered thermogenesis (Blankenhaus et al., 2019), we also found that the hematopoietic system was severely compromised because CMPs, GMPs, and CLPs in *Fth*^*Δ/Δ*^ mice were reduced significantly in comparison with *R26*^*Cre*^ and *Fth*^*fl/fl*^ control mice (Figures 6B, 6C, and S6A). This suggests that FTH is essential to sustain hematopoiesis. To overcome the short life expectancy of *Fth*^*Δ/Δ*^ mice, we generated BM chimeric mice in which FTH is deleted specifically in the hematopoietic compartment, allowing us to investigate the role of FTH in hematopoietic cells. Lethally irradiated WT (CD45.1^+^) mice reconstituted with ROSA26^Cre^ER^T2^Fth^lox/lox^ BM (CD45.2^+^) received tamoxifen (BM-*Fth*^*−/−*^) or vehicle (BM-*Fth*^*+/+*^) for 5 days (Figures 6D and 6E). Similar to *Mtb-*infected or poly(I:C)-treated mice, in BM-*Fth*^*−/−*^ mice, the frequency and total number of CMPs (Figure 6F) were decreased significantly, which correlated with reduced mitochondrial Fe^2+^ (Figure 6G) and mitochondrial depolarization (Figure 6H). However, the frequency, but not total number, of CLPs was increased significantly (Figure S6B), and no alterations in mitochondrial Fe were observed (Figure S6C). Strikingly, this dysregulation of myelopoiesis in BM-*Fth*^*−/−*^ mice had a substantial effect on anti-mycobacterial responses because BMDMs from BM-*Fth*^*−/−*^ mice failed to control the growth of virulent *Mtb* but not avirulent BCG (Figures 6I and S6D). This was relevant *in vivo* because, similar to mice lacking FTH in myeloid cells (Reddy et al., 2018), BM-*Fth*^*−/−*^ mice were severely susceptible to aerosol *Mtb* infection, exhibiting significantly increased *Mtb* growth in the BM, lungs, liver, and spleen (Figures 6J, 6K, S6E, and S6F) and succumbing to death within 50 days (Figure 6L). This occurred despite largely normal myeloid cell frequencies in the lungs, BM, and spleen prior to infection (Figures S6G–S6I). These results collectively indicate that dysregulation of the IFN-I/iron axis severely impairs myelopoiesis and causes susceptibility to *Mtb* infection.

### Mycobacterium-Mediated Functional Rewiring of BM-HSCs and Progenitors Is Maintained up to at Least 1 Year

**Figure 7:**
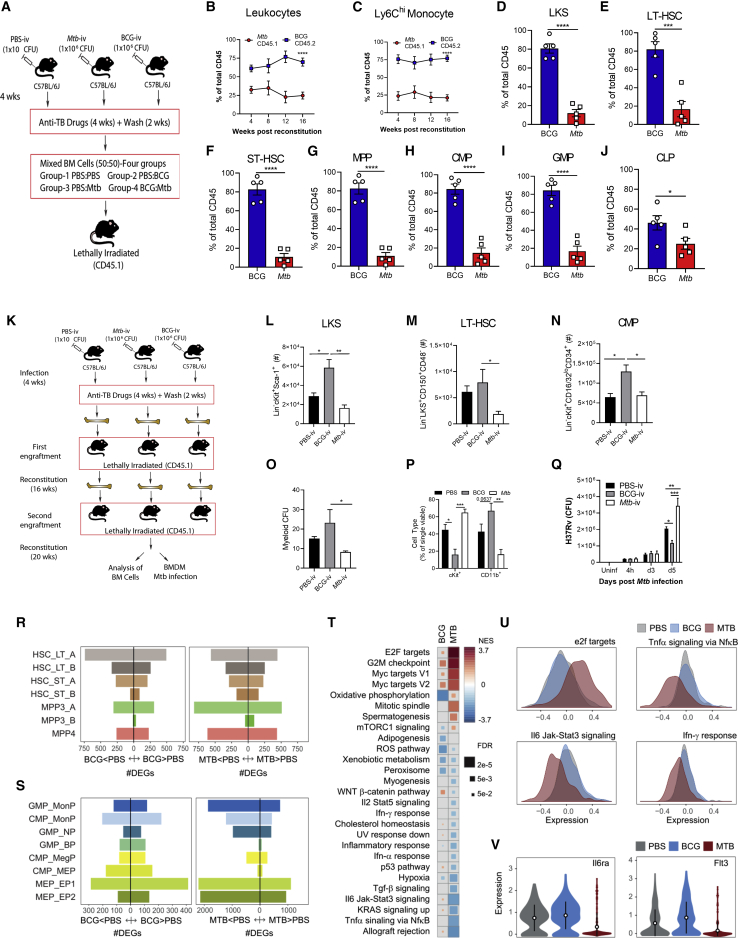
*Mtb-*i.v. Causes BM Exhaustion and Has Long-Term Effects on BMDM Training (A) Mixed chimera model. (B and C) Percentage of CD45.1^+^ versus CD45.2^+^ leukocytes (B) or Ly6C^hi^ monocytes (C) in the blood (n = 5–10/group). (D–J) In the BM, percentage of LKS cells (D), LT-HSCs (E), ST-HSCs (F), MPPs (G), CMPs (H), GMPs (I), and CLPs (J) after 16 weeks of reconstitution. (K) Secondary engraftment model. (L–N) Number of LKS cells (L), LT-HSCs (M), and CMPs (N). (O and P) Myeloid colonies (O) and cell type by flow cytometry (P) by BM methylcellulose CFU assay. (Q) *In vitro* BMDM CFUs. (R and S) Number of genes up- and downregulated by *Mtb* and BCG in each cluster (abs(logFC) > 0.1 and FDR < 0.05) of LKS cells (R) and cKit^+^ cells (S). (T) GSEA of genes ranked according to BCG (left) or *Mtb* (right) effects in the GMP_MonP cluster. (U) Distribution of expression levels in GMP_MonP for gene set average markers in four different hallmark gene sets. (V) Il6ra and Flt3 expression in the GMP_MonP cluster. Two-way ANOVA followed by Sidak’s multiple comparisons test was used in (B), (C), (P), and (Q). One-way ANOVA followed by Tukey’s multiple comparisons test was used in (L)–(O). In (D–J), two-tailed Student’s t test was used to assess differences. In (B) and (C), only significant differences at 16 weeks are depicted. See also Figure S7.

**Figure S7:**
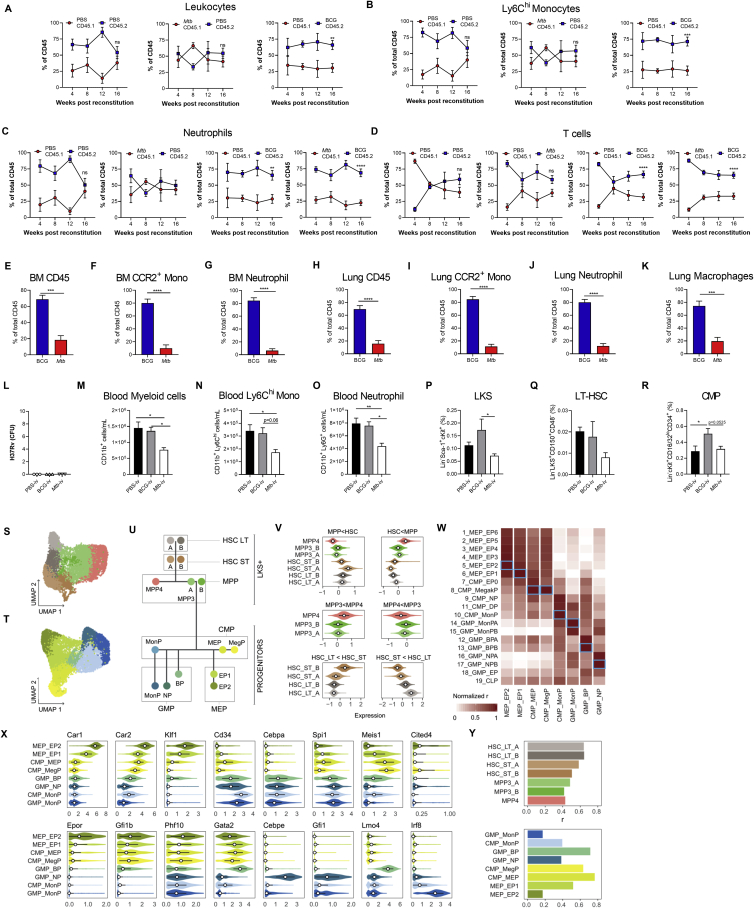
*Mtb*-Imprinted HSCs Have Impaired Engraftment for up to at Least 1 Year Post-exposure, Related to Figure 7 (A-K) Mixed chimeric mice were generated as described in Figure 7A. At 4-week intervals post-reconstitution, peripheral blood was sampled. Percentages of CD45.1^+^ versus CD45.2^+^ leukocytes (A), Ly6C^hi^ monocytes (B), neutrophils (C) or T cells (D) in the blood. At 16 weeks post-reconstitution, BCG:*Mtb* mixed chimera mice were sacrificed. Frequency of total BM CD45 (E), CCR2^+^ monocytes (F), or neutrophils (G), as well as pulmonary leukocytes (H), CCR2^+^ monocytes (I), neutrophils (J) and macrophages (K). (L-R) Secondary engraftment experiments were performed as in Figure 7K. *Mtb* CFU in the BM cells prior to secondary engraftment (L). In the peripheral blood, total cell counts of CD11b^+^ cells (M), Ly6C^hi^ monocytes (N) and neutrophils (O), as well as BM LKS (P), LT-HSC (Q) and CMP (R) frequencies. (S-T) UMAP dimensionality reduction plots for LKS and myeloid progenitor cells, respectively, for the serial engraftment experiment. (U) Schematic hematopoietic tree diagram showing the approximated position of the different clusters identified (cluster specific color code common to panels S-T). (V) Average expression of cell-type markers across clusters. (W) Correlations between genome-wide expression patterns of our myeloid progenitor data and results published by Paul et al. (2015). In each column, Spearman correlations are normalized to cover the range [0-1]. Blue boxes mark the best fit (i.e., candidate identity match) for each of our clusters. (X) Expression patterns across clusters for some marker genes associated to commitment to the different lineages characterized in this study. (Y) Genome wide correlation of *Mtb* versus BCG effect sizes (logFC) in each cluster. Differences measured via Two-Way ANOVA followed by Sidak’s Multiple Comparison Test (A-D), Student’s Two-tailed T-Test (E-K) and One-Way ANOVA followed by Tukey’s Multiple Comparisons Test in (M-R). In A-D, only significant differences at 16 weeks were labeled.

Our results indicate that BCG reprograms HSCs, ultimately leading to trained immunity and increased protection against TB. In contrast, *Mtb* induces regulatory changes in HSCs that fail to induce trained immunity and lead to BMDMs with impaired control of *Mtb*. HSC exhaustion in the BM has been reported in human TB patients since the 1980s (Hunt et al., 1987), but how *Mtb*/BCG may modulate the long-term (LT) functional capacity of HSCs is unknown. To directly compare the reconstitution capacity of mycobacterium-exposed BM, we performed a competitive mixed chimera experiment. Lethally irradiated mice were reconstituted with mixed BM cells (50/50) of *Mtb* (CD45.1^+^), BCG (CD45.2^+^), or PBS (either CD45.1^+^ or CD45.2^+^) and treated with antibiotics for 4 weeks to eliminate mycobacteria and then rested for 2 weeks (Figure 7A). Beginning at 4 weeks and continuing every 4 weeks until 16 weeks post-reconstitution, when LT-HSC-dependent hematopoiesis is established, we analyzed the percentage of CD45.1^+^ and CD45.2^+^ cells in peripheral blood. At 16 weeks, in the PBS:PBS group, the frequencies of CD45.1^+^ compared with CD45.2^+^ donor cells in all leukocyte populations tested were equal (Figures S7A–S7D, left panels), excluding any potential artificial bias in engraftment of CD45.1^+^ cells compared with CD45.2^+^ cells. However, although the proportion of circulating leukocytes was comparable in the *Mtb*:PBS group (Figures S7A–S7D, second panel), the BCG:PBS and BCG:*Mtb* groups showed a significant bias to be derived from BCG-i.v. mice (Figures 7B, 7C, S7A, and S7B, right panel, and S7C and S7D, third and fourth panels). This was due to superior engraftment of BCG-exposed HSCs because at 16 weeks post-reconstitution, BCG-derived HSCs and progenitors as well as effector leukocytes in the BM and lungs, dominated BCG:*Mtb* chimeras (Figures 7D–7J and S7E–S7K). Thus, BCG-exposed HSCs exhibit superior engraftment compared with control or *Mtb*-exposed HSCs, indicating that BCG reprograms HSCs to enhance their functional capacity.

To further explore the durability and length of HSC imprinting following exposure to mycobacteria, we next employed a serial engraftment model. CD45.2^+^ mice were infected with *Mtb*, BCG, or PBS control for 4 weeks, treated with anti-mycobacterial drugs for 4 weeks, and rested for 2 weeks. BM from each group was transplanted into lethally irradiated CD45.1^+^ mice (primary engraftment). After 16 weeks, BM was re-harvested and transplanted into a second group of lethally irradiated CD45.1^+^ mice (secondary engraftment) (Figure 7K). After 20 weeks, no mycobacteria could be found in the BM (Figure S7L), and there was a significant reduction in total myeloid cells in the blood as well as Ly6C^hi^ monocytes and neutrophils in the recipients of *Mtb-*i.v. BM (Figures S7M–S7O). In line with the reduced leukocytes in the circulation, the HSC compartment of the recipients of *Mtb*-i.v. BM had fewer LKS cells, LT-HSCs, and CMPs (Figures 7L–7N and S7P–S7R). Additionally, we incubated BM cells from each group in MethoCult medium to assess the hematopoietic output potential. Mice that received BM from the BCG-i.v. group produced more colonies and mature CD11b-expressing myeloid cells (as determined by flow cytometry) than mice that received *Mtb*-i.v. BM (Figures 7O and 7P). Having determined the impaired function of *Mtb*-exposed HSCs, we then sought to elucidate the longevity of trained immunity. We derived BMDMs from secondary engraftment mice and infected them with *Mtb in vitro.* Remarkably, after two reconstitutions and almost a year after the initial mycobacterium exposure, BMDMs from BCG-i.v. mice still provided significant protection from *Mtb*, whereas BMDMs from *Mtb*-i.v. mice continued to exhibit impaired control (Figure 7Q).

To examine the training program of BM-HSCs and progenitors after secondary engraftment, we performed scRNA-seq on HSCs and progenitor cells from animals that had originally been exposed to BCG, *Mtb*, or PBS to evaluate whether there were any transcriptional differences still present. After QC, we kept a total of 7,959 LKS cell transcriptomes (3,872 PBS, 2,524 BCG, and 1,563 *Mtb*) and 9,859 transcriptomes from cKit^+^ cells (3,353 PBS, 5,020 BCG, and 1,486 *Mtb*). These cells were clustered in 7 and 8 groups in the LKS cell and progenitor populations, respectively (Figures S7S–S7U; see STAR Methods and Figures S7V–S7X for details regarding LKS and progenitor clustering inference and validation). In the LKS population, we observed 2,684 and 2,230 DEGs between PBS and *Mtb* or BCG in at least one of the cell clusters (Figure 7R; Tables S5 and S6). In progenitors, the effects of *Mtb* versus PBS (5,574 DEGs) appeared to be much more pronounced than BCG versus PBS (1,392 DEGs) (Figure 7S). Genes that respond significantly to infection (FDR < 0.05 and abs(logFC) > 0.1 for BCG-PBS or *Mtb*-PBS) show highly correlated treatment effects (Pearson correlation averaged across clusters, r = 0.53 and 0.47 for LKS cells and progenitor populations, respectively; p < 1E−18 in all clusters; Figure S7Y). Interestingly, among all cell clusters identified, the most committed MonPs (GMP_MonPs) showed the most divergent response between BCG and *Mtb*, possibly explaining their remarkable differences in BMDM control of *Mtb* growth (Figure 7Q). To further explore the pathways regulated differentially in MonPs between the two groups, we performed GSEA on the *Mtb* and BCG effects in this cluster. Focusing on the hallmark list of gene sets (Liberzon et al., 2015), we detected 27 gene sets significantly enriched among DEGs between BCG-i.v. or *Mtb*-i.v. and control conditions (Figure 7T; FDR < 0.01; STAR Methods), with most pathways enriched uniquely under BCG-i.v. or *Mtb*-i.v. conditions. To explicitly dissect the divergence in the regulatory pathways altered by BCG-i.v. and *Mtb*-i.v., for every MonP cell, we calculated the average gene expression of all genes annotated in each of the sets analyzed. We then compared differences in the overall activity of each of the pathways between MonP cells derived from each group (Figure 7U). These analyses revealed that monocyte progenitors from *Mtb*-i.v. mice present a phenotype that is characterized by impaired inflammatory activity, as shown by the coordinated decrease in the expression levels of genes associated with regulation of inflammatory responses as well as IFN-I and IFN-II. In contrast, genes involved in the E2F pathway, which is a critical regulator of cell proliferation versus apoptosis (Polager and Ginsberg, 2009), showed increased activity in monocyte progenitors from *Mtb*-i.v. compared with BCG-i.v. mice. Consistent with these findings, Gene Ontology enrichment analyses for the set of DEGs between monocyte progenitors from BCG-i.v. and *Mtb*-i.v. (1,707 under a more stringent cutoff: abs(logFC) > 0.2 and FDR < 0.01) revealed that genes upregulated in BCG-i.v. compared with *Mtb*-i.v. were significantly enriched in terms related to regulation of cytokine production, such as IFN-I (FDR = 1.5E−4) or IL-6 (FDR = 2.6E−5). In turn, genes more highly expressed in *Mtb*-i.v. than BCG-i.v. were enriched in functions related to regulation of cell proliferation, such as mitotic nuclear division (FDR = 2.1E−23) as well as terms related to mitochondrial respiration, such as oxidative phosphorylation (FDR = 8.1E−17), the ATP biosynthesis process (FDR = 2.9E−8), and the respiratory electron transport chain (FDR = 6.5E−10) (see Table S7 for the complete results). Key genes for regulation of hematopoiesis (Flt3) and deployment of inflammatory responses (Il6ra) appear to be regulated divergently in *Mtb* versus BCG (Figure 7V). Collectively, these results demonstrate that the effect of BCG and *Mtb* on the transcriptional profile of HSCs and progenitors contributes to protective or failed trained immunity, respectively, and lasts for at least 1 year.

## Discussion

One of the hallmarks of chronic infection is impaired immune-driven resistance, allowing pathogen persistence (Altare et al., 1998; Divangahi et al., 2008). Given that HSCs generate all non-embryonically seeded immune cells, pathogen manipulation of HSC responses will affect host defense to infection. Although some mechanisms of rapid HSC adaptation to acute infection (e.g., *E. coli*) have been studied, our understanding of the HSC response to chronic infection, including *Mtb,* is extremely limited. Interestingly, BM suppression in TB was documented in the late 1980s (Hunt et al., 1987), and it has been shown that *Mtb* persists in the BM by exploiting mesenchymal stem cells as a niche for its survival (Das et al., 2013). However, the effect of *Mtb* on HSC function and the subsequent immune response are incompletely understood. Here we demonstrate that *Mtb* accesses the BM as early as 10 days after pulmonary infection and reprograms HSCs at two critical levels: (1) depleting myeloid progenitors and (2) impairing trained immunity, both of which compromise host resistance to infection.

We recently showed how BCG and adjuvants like β-glucan provide protective trained immunity against *Mtb* via HSC reprogramming (Kaufmann et al., 2018; Moorlag et al., 2020). Although there is a striking difference in the pathogenesis of BCG versus *Mtb* (Figure 1B), with the exception of an early time point (day 7), the absolute numbers of HSC and MPP populations remained similar. However, BCG and *Mtb* were highly dichotomous in their modulation of lineage-restricted progenitors, where *Mtb* specifically inhibited myelopoiesis. It was initially thought that specific transcriptional networks of individual HSCs were the major determinants for driving specific lineages (Kee, 2011; Novershtern et al., 2011). However, similar to a recent study (Lauridsen et al., 2018), we found that the transcriptional networks for myelopoiesis or lymphopoiesis were similar in HSCs/MPPs after BCG vaccination or *Mtb* infection. Rather, the magnitude of several key pathways, including IFN-I and heme/iron metabolism, differed considerably. Both were implicated in lineage commitment downstream of HSCs/MPPs because of the susceptibility of myeloid but not lymphoid progenitors to *Mtb*-induced necroptosis, as has been shown similarly during shock-like *Ehrlichia* infection (Smith et al., 2018). Additionally, we found that IFN-I signaling alters mitochondrial Fe levels, disrupts MMP, and drives necroptosis specifically in CMPs/GMPs. Our observation that IFN-I was responsible for CLP expansion in poly (I:C) but not *Mtb* is likely due to the fact that poly(I:C) specifically triggers IFN-I production acutely, whereas *Mtb* chronically activates many pathways that can promote lymphopoiesis independent of IFN-I signaling.

Over 80% of available Fe in mammals exists in the form of heme, and therefore pathogenic microorganisms likely evolved to re-direct Fe as well as heme from their hosts (Soares and Weiss, 2015). Fe plays a central role in TB pathogenesis because it is required for growth of *Mtb* (Marcela Rodriguez and Neyrolles, 2014; Reddy et al., 2012). Indeed, mice lacking the heme-catabolizing enzyme heme oxygenase-1 (HO-1) are highly susceptible to *Mtb* infection (Regev et al., 2012; Silva-Gomes et al., 2013). The Fe generated via heme catabolism is primarily stored in its inert ferric form by the protein ferritin (Gozzelino and Soares, 2014). Similar to HO-1-deficient mice, we found that BM-*Fth*^*−/−*^ mice showed a reduction of myelopoiesis at steady state and were highly susceptible to *Mtb* infection. Although we have not directly linked Fe metabolism to specific cell death programs, the increased expression of RIPK3 in HSCs, the involvement of necroptosis in BM failure (Rickard et al., 2014; Roderick et al., 2014; Smith et al., 2018), the unique evolutionary link between *Mtb* and necroptosis (Behar et al., 2010; Divangahi et al., 2009; Roca and Ramakrishnan, 2013; Zhao et al., 2017), and the enhanced viability of *Ripk3*^*−/−*^ myeloid progenitors following *Mtb* infection suggest that the IFN-I/iron axis biases hematopoiesis via necroptosis. Our results are also compatible with an elegant study showing that, although tumor necrosis factor (TNF) promotes cell death in myeloid progenitors, it prevents HSC necroptosis (Yamashita and Passegue, 2019). Therefore, there are safeguard mechanisms in HSCs that are absent in more committed myeloid progenitors.

Adjuvants such as β-glucan via IL-1 signaling (Moorlag et al., 2020) or live vaccines like BCG via IFN-II (Kaufmann et al., 2018) sustain myelopoiesis with protective imprinting to generate trained immunity. However, *Mtb* limits myelopoiesis and impairs trained immunity in an IFN-I-dependent manner. Although the hematopoietic system rapidly adapts to stress to meet the demand of the immune response, this on-demand hematopoiesis is context specific with unique imprinting and lineage fate decisions. For instance, IFN-I therapies are rarely associated with BM suppression (Ioannou et al., 2010; Platanias and Fish, 1999), indicating a that second stimulus is required to cause BM failure. During LCMV infection, the suppressive effects of IFN-I on hematopoiesis require IFN-II (de Bruin et al., 2013). Given that the levels of IFN-II gene expression in HSCs and progenitor cells are similar in BCG and *Mtb* infection, it is tempting to speculate that during *Mtb* infection, IFN-I may override the protective effects of IFN-II in BCG-vaccinated mice so that the relative magnitudes of these signals on HSCs determine a protective versus detrimental response to infection.

One of the outstanding questions in the field of trained immunity is the longevity of imprinting in trained immune cells. Using a serial engraftment approach, we demonstrated that imprinting of HSCs by BCG or *Mtb* lasts for at least a year and is transmitted to macrophages. Additionally, our competitive mixed chimera experiments indicate that BCG enhances the capacity of HSC engraftment, an important consideration for clinical management of TB and BM transplantation. Of particular clinical interest is our observation that aerosolized *Mtb* accesses the BM and reprograms HSCs similarly to the systemic model of TB. While the majority of studies in mice and humans have focused on the pulmonary response, our results show that *Mtb* disseminates from the lungs to the BM by 10 days post-infection. Although our transcriptomics data are at 120 days post-infection, the early presence of *Mtb* suggests that reprogramming may begin more rapidly, and future studies should focus on discerning the kinetics of this imprinting in the preclinical and clinical settings.

### Limitations of Study

Although our study provides intriguing observations regarding reprogramming of HSCs to generate protective or impaired innate immunity against TB, at least three outstanding issues remain:

(1) Genomics. Although we show that the longevity of mycobacterial imprinting lasts for at least 1 year, the mechanisms of how HSCs are able to maintain these signatures in the complete absence of mycobacteria for this lengthy period of time are still unknown. Thus, studies investigating the LT epigenetic landscape and transcriptomes of HSCs versus BMDMs following mycobacterium exposure are required to address this important question.
(2) Host-pathogen interaction. Our results indicate that the effect of IFN-I on Fe metabolism could be a strategy for *Mtb* to obtain this essential element for its growth. However, we know little about the genomic region (e.g., RD1) or virulence factors of *Mtb*, which are responsible for modulating the IFN-I/Fe axis in the BM. Why myeloid progenitors, but not HSCs or lymphoid progenitors, are prone to death during *Mtb* infection and how exactly Fe metabolism is linked to necroptosis also remain to be determined.
(3) TB pathogenesis. Because *Mtb* biases HSCs toward lymphopoiesis, it may also intrinsically reprogram lymphoid lineages to generate ineffective lymphocytes against TB. Although this was not explored in our study, it may explain why there is no natural immunity to TB and the lack of association between increased conventional T cell responses and protection against *Mtb* (Barber et al., 2011; Nemes et al., 2018; Tameris et al., 2013; Tzelepis et al., 2018). This type of immune subversion is conceptually similar to the observation that human T cell epitopes of *Mtb* are evolutionarily hyperconserved (Comas et al., 2010) and the paradoxical notion that T cells may actually contribute to TB transmission by participating in induction of cavitary lung disease (Genewein et al., 1993; Rodrigo et al., 1997). Certainly, future studies investigating the potential effect of *Mtb* on lymphopoiesis are required to carefully test this hypothesis.

## STAR★Methods

### Key Resources Table

**Table:** 

REAGENT or RESOURCE	SOURCE	IDENTIFIER
**Antibodies**		

Fixable Viability Stain eFluor501	eBioscience	Cat#65-0863-18
anti-CD16/32 (clone 93)	eBioscience	RRID: AB_467134
anti-Ter-119 biotin-conjugated (clone Ter119)	BD Bioscience	RRID: AB_394985
anti-CD11b biotin-conjugated (clone M1/70)	BD Bioscience	RRID: AB_394773
anti-CD5 biotin-conjugated (clone 53-7.3)	BD Bioscience	RRID: AB_394557
anti-CD4 biotin-conjugated (clone RM4-5)	BD Bioscience	RRID: AB_394581
anti-CD8a biotin-conjugated (clone 53-6.7)	BD Bioscience	RRID: AB_394567
anti-CD45R biotin-conjugated (clone RA3-6B2)	BD Bioscience	RRID: AB_394616
anti-Ly6G/C biotin-conjugated (clone RB6-8C5)	BD Bioscience	RRID: AB_394641
Streptavidin – APC-Cy7	eBioscience	RRID: AB_10366688
Streptavidin - BUV395	BD Bioscience	Cat# 564176
anti-c-Kit – APC (clone 2B8)	eBioscience	RRID: AB_469430
Anti-cKit-BV421 (clone 2B8)	BD Bioscience	RRID: AB_2739664
anti-Sca-1 – PE-Cy7 (clone D7)	eBioscience	RRID: AB_469668
anti-CD150 – eFluor450 (clone mShad150)	eBioscience	RRID: AB_2574045
anti-CD48 – PerCP-eFluor710 (clone HM48-1)	BD Bioscience	RRID: AB_396724
anti-Flt3 – PE (clone A2F10.1)	BD Bioscience	RRID: AB_395079
anti-CD34 – FITC (clone RAM34)	eBioscience	RRID: AB_465020
Anti-CD16/32-PerCP efluor 710 (clone 93)	eBioscience	RRID: AB_996659
Anti-CD115 - BV711 (clone AFS98)	Biolegend	RRID: AB_2562679
anti-F4/80 – APC-Cy7 (clone BM8)	eBioscience	RRID: AB_469452
Anti-F480-APC (clone BM8)	eBioscience	RRID: AB_2784647
anti-Ly6C – APC (clone HK1.4)	eBioscience	RRID: AB_1724155
anti-Ly6C – FITC (clone AL-21)	BD Bioscience	RRID: AB_394628
Anti-Ly6C – APC (clone HK1.4)	eBioscience	RRID: AB_1724153
anti-Ly6G – PerCP-eFluor710 (clone 1A8-Ly6g)	eBioscience	RRID: AB_2573892
anti-Siglec F – PE-CF594 (clone E50-2440)	BD Bioscience	RRID: AB_2687994
anti-CD11c – PE-Cy7 (clone HL3)	BD Bioscience	RRID: AB_2033997
anti-CD11b – eFluor 450 (clone M1/70)	BD Bioscience	RRID: AB_1582236
anti-NK1.1 – BV650 (clone PK136)	BD Bioscience	Cat#564143
anti-CD19 – PE-Cy7 (clone 1D3)	eBioscience	RRID: AB_657664
anti-CD3 – PE (clone 145-2C11)	BD Bioscience	RRID: AB_465496
anti-CD4 – FITC (clone GK1.5)	eBioscience	RRID: AB_464892)
anti-CD4 - eFluor 450 (clone GK1.5)	eBioscience	RRID: AB_10718983
anti-CD8 – AlexaFluor 700 (clone 53-6.7)	BD Bioscience	RRID: AB_396959
anti-CD127 – BV786 (clone SB/199)	BD Bioscience	RRID: AB_2738403
Anti CD127 - BV605 (clone A7R34)	Biolegend	RRID: AB_2562114
anti-Ly6G – AlexaFluor700 (clone RB6-8C5)	eBioscience	RRID: AB_10611860
anti-CD45.1 – APC (clone A20)	BD Bioscience	RRID: AB_1645214
anti-CD45.2 – BUV395 (clone 104)	BD Bioscience	Cat# 564616
anti-FTH	Cell Signaling Technology	RRID: AB_11217441
anti-CD71-eFluor 450 (clone R17217)	eBioscience	RRID: AB_2574027)
Anti-Ki67 PE (clone 16A8)	BioLegend	RRID: AB_2561524
MACS Streptavidin-conjugated microbeads	Miltenyi Biotec	Cat#130-048-101
DAPI	Sigma	Cat#D9542
MethoCult	Stem Cell Technologies	Cat# GF M3534
rhodamine B 4-[(1,10-phenanthrolin-5-yl)aminocarbonyl]benzyl ester (RPA)	Squarix Biotechnology	Cat#ME043.1
MitoTracker Green FM	ThermoFischer Scientific	Cat#M7514
MitoTracker Orange CMTMRos	ThermoFischer Scientific	Cat#M7510
MitoSOX Red	ThermoFischer Scientific	Cat# M36008
Viability NucSpot Far-Red	Biotium	Cat#40085
PE Annexin V Apoptosis Detection Kit with 7-AAD	BioLegend	Cat# 640934
FOXP3 Transcription Factor Staining Kit	eBioscience	Cat# 00-5523-00
B16-Blue IFN-α/β Cells	Invivogen	Cat# bb-ifnt1

**Bacterial and Virus Strains**		

*Mycobacterium bovis* BCG-TICE (TMC 1028)	ATCC	ATCC Number: 35743
*Mycobacterium tuberculosis* H37Rv (TMC 102)	ATCC	ATCC Number: 27294
*Mycobacterium bovis* BCG-GFP	Dr. Marcel Behr, McGill University, Montreal	Alter et al., 2010
*Mycobacterium tuberculosis* H37Rv-GFP	Dr. Marcel Behr, McGill University, Montreal	This Paper
Mycobacterium tuberculosis H37Rv-ΔRD1	Dr. Marcel Behr, McGill University, Montreal	Lewis et al., 2003

**Chemicals, Peptides, and Recombinant Proteins**		

Poly (I:C) (LMW)	Invivogen	Cat#tlrl-picw
β-Glucan	Sigma-Aldrich	Cat#G5011
Isoniazid	Sigma-Aldrich	Cat#13377-150G
Rifampicin	EMD Millipore	Cat#557303-5GM
Enrofloxacin, Baytril 50mg/ml	Bayer	DIN 02169428
RPMI-1640 with L-glutamine	Wisent	Cat#350-000CL
Middlebrook 7H9 broth	Fischer Scientific	Cat#CDF0713-17-9
Middlebrook 7H10 Agar	Fischer Scientific	Cat#DF0627-17-4
Glycerol for ADC	Wisent	Cat#800-0400-LL
BSA for ADC	Wisent	Cat#800-195-EG
Dextrose for ADC	Fisher Scientific	Cat#D16-10 CAS Number 50-99-7
NaCl for ADC	Fischer Scientific	Cat#S6713
Oleic acids for OADC	Sigma	Cat#364525
Tween 80	Fischer Scientific	Cat#338-500 CAS Number 9005-65-6
FBS	Wisent	Cat#80150
HEPES	Wisent	Cat#330-050-EL
Non-Essential Amino acids	Wisent	Cat#321-010-EL
Essential Amino acids	Wisent	Cat#321-011-EL
Sodium Pyruvate	Wisent	Cat#600-110-EL
Penicillin/Streptomycin	Wisent	Cat#450-115-EL
Corning Cell Stripper	Fischer Scientific	Cat#MT25056Cl
Collagenase IV	Sigma	Cat#C5138
Paraformaldehyde 1%	Thermo Fischer	Cat#28908
Kanamycin	Wisent	Cat#400-145-1G
QIAzol Lysis Reagent	QIAGEN	Cat#79306
RT Mastermix	Diamed	Cat#ABMG490
Abm EvaGreen	Diamed	Cat#ABMMastermix-S
PANTA	Fischer Scientific	Cat#B4345114

**Critical Commercial Assays**		

Magmax-96 Total RNA Isolation Kit	ThermoFischer	Cat#AM1830
SignalFire ECL Reagent	Cell Signaling Technologies	Cat#6883
Tamoxifen	Sigma-Aldrich	Cat #T5648

**Deposited Data**		

Bulk RNA-Seq, Single cell RNA-Seq	Gene expression ómnibus (GEO) database	GSE156137

**Experimental Models: Cell Lines**		

L929 cell line	ATCC	ATCC Number: CCL-1
B16-Blue IFN-α/β Cells	InvivoGen	Cat#bb-ifnt1

**Experimental Models: Organisms/Strains**		

Mouse:C57BL/6J	The Jackson Laboratory	IMSR Cat# JAX:000664, RRID: IMSR_JAX:000664
Mouse: *Ifnar1−/−*	The Jackson Laboratory	IMSR Cat# JAX:028288
Mouse: B6.129S7-Ifngr1tm1Agt/J	The Jackson Laboratory	IMSR Cat# JAX:003288, RRID: IMSR_JAX:003288
Mouse CD45.1+ B6. SJL-Ptprca Pepcb/BoyJ	The Jackson Laboratory	IMSR cat# JAX:002014, RRID: IMSR_JAX:002014
Mouse: Rosa26^Cre^ER^T2^Fth^lox/lox^	Dr. MP Soares (Instituto Gulbenkian Ciencia, Portugal)	(Blankenhaus et al., 2019)
Mouse*: Ripk3−/−*	Dr. Vishva Dixit (Genentech, San Francisco)	(Newton et al., 2014)
Mouse: NOD.129S7(B6)-Rag1tm1Mom/J	The Jackson Laboratory	IMSR Cat# JAX:003729, PRID: IMSR_JAX003729

**Software and Algorithms**		

Graph Pad Prism, version 6.0c	http://www.graphpad.com/scientific-software/prism/	SCR_015807
FACSDiva Software	BD Biosciences	SCR_001456
FlowJo software v.10.1	Tree Star	SCR_000410
Amnis INSPIRE software	Luminex	https://www.luminexcorp.com/imaging-flow-cytometry/
Amnis IDEAS software	Luminex	https://www.luminexcorp.com/imaging-flow-cytometry/
Trim Galore (version 0.2.7)	Krueger, n.d.	http://www.bioinformatics.babraham.ac.uk/projects/trim_galore/
edgeR	Robinson et al., 2010	SCR_012802
limma	Ritchie et al., 2015	SCR_010943
CLUEGO	Bindea et al., 2009	SCR_005748
kallisto (v0.43.0)	Bray et al., 2016	
Seurat	Satija et al., 2015	SCR_007322

### Resource Availability

#### Lead Contact

Further information and requests for resources and reagents should be directed to and will be fulfilled by the Lead Contact, Maziar Divangahi (maziar.divangahi@mcgill.ca).

#### Materials Availability

No unique reagents were generated for this study.

#### Data and Code Availability

All data generated in this study (bulk and single cell RNA-Seq) are available in GEO database under the accession number GEO: GSE156137. All the Software packages and methods used in this study have been properly detailed and referenced under “Quantification and Statistical Analysis.”

### Experimental Model and Subject Details

#### Mice

Six- to ten-week old CD45.1 and CD45.2 C57BL/6J, *Ifnar1*^−/−^, and *Ripk3*^*−/−*^ (kindly provided by Dr. Vishva Dixit, Genentech) mice were housed and bred at the RI-MUHC, Montreal, QC, Canada. Rosa26^Cre^ER^T2^Fth^lox/lox^ mice were provided by MP Soares (Portugal) and tamoxifen treatment was performed as previously described (Blankenhaus et al., 2019). All animal studies were conducted in accordance with the guidelines of, and approved by, the Animal Research Ethics Board of McGill University (project ID: 5860). Mice were housed under SPF conditions with *ad libitum* access to food and water. Experiments were conducted using male and female sex- and age-matched mice that were randomly assigned to experimental groups.

### Method Details

#### Bacterial culture

*Mtb* H37Rv, *Mtb* H37Rv ΔRD1 (Lewis et al., 2003) and BCG-TICE were grown in 7H9 broth (BD) supplemented with 0.2% glycerol (Wisent), 0.05% Tween80 (Fisher), and 10% albumin-dextrose-catalase (ADC) under constant shaking at 37°C. For experiments involving *Mtb*-GFP and BCG-GFP, bacteria were grown as above with the addition of kanamycin (25μg/mL; Wisent) for selection of resistant bacteria. For *in vitro* and *in vivo* experiments, *Mtb* (H37Rv) bacteria in log growing phase (OD 0.4 – 0.9) were centrifuged (4000 RPM, 15 minutes) and resuspended in RPMI without penicillin/streptomycin or sterile PBS. Single cell suspensions were obtained by passing the bacteria 10-15 times through a 22G needle (Terumo).

#### *In vivo* experiments

##### Mtb infection or BCG vaccination of mice

Throughout the study, mice were intravenously (iv) infected with *Mtb* H37Rv, *Mtb* H37Rv-ΔRD-1 or vaccinated with BCG-TICE with a dose of 1x10^6^ single-suspended bacteria in 100μl PBS, unless otherwise indicated. For aerosol infection, mice were infected with approx. 50-100 CFU of *Mtb* H37Rv in a nose-only aerosol exposure unit (Intox). Infection dose was confirmed by enumerating the lung CFU one-day after infection.

##### Antibiotic treatment of mice

For antibiotic treatment with antimycobacterial drugs, the drinking water of the mice was supplied with 100mg/L Isoniazid (INH; Sigma-Aldrich) and 100mg/L Rifampicin (RIF; Fisher) for four weeks. The antibiotic treatment was followed by a two-week wash-out period on regular water.

##### Poly (I:C) and β-Glucan treatment

C57BL/6J and *Ifnar1*^*−/−*^ mice were treated with Poly (I:C) (Invivogen) (200μg/mouse) intraperitoneally (i.p.) at day 0, 2, 4, 6. Mice were sacrificed at day 3 and day 7 depending on the experiment. For β-glucan (Sigma, catalog #G5011), mice were treated with 1mg/mouse i.p. at day 0 and day 3. Mice were sacrificed at day 7.

##### Adoptive transfer experiment

C57BL/6J mice were vaccinated with BCG-iv or infected with Mtb-iv (1x10^6^ CFU) or PBS injected (PBS-iv). After 4 weeks, BM cells were harvested and differentiated into BMDMs in the presence of anti-TB drugs to kill any residual *Mtb*. BMDMs were infected with H37Rv (MOI of 0.2) for 30 minutes at 37°C and 5% CO2 with frequent agitation. Free bacteria were then removed by washing 5x with cold RPMI, each followed by centrifugation (1500 RPMI) for 10 min at 4°C. BMDMs (0.5x10^6^ cells) were resuspended in 40 μL PBS and then intratracheally transferred into naive *Rag1*^*−/−*^ mice (Divangahi et al., 2009, Divangahi et al., 2010). The initial number of bacteria prior to transfer was assessed by plating *Mtb*-infected macrophages on 7H10 agar plates.

##### Competitive Mixed chimeric mouse model

C57BL/6J mice were either PBS-injected or BCG iv-vaccinated or *Mtb* iv-infected (1x10^6^ bacteria). After four weeks, mice were treated with anti-TB drugs for four weeks. The antibiotic treatment was followed by a two-week wash-out period on regular water. BM cells (2x10^5^/group) from various pairs of either BCG iv-vaccinated, *Mtb*-iv infected or PBS controls were admixed and intravenously injected into CD45.1^+^ recipient C57BL/6J mice 16 hours post lethal irradiation with 9 Gy. Mice were kept under antibiotic treatment (0.5g Enrofloxacin (Bayer) per liter of drinking water) for three days before and two weeks after irradiation along with anti-TB drugs for 5 days. Engraftment was measured after 4, 8, 12 and 16 weeks of reconstitution by staining peripheral blood cells.

##### Secondary engraftment mouse model

C57BL/6J mice were either PBS-injected or BCG iv-vaccinated or *Mtb* iv-infected (1x10^6^ bacteria). After four weeks, mice were treated with anti-TB drugs for four weeks. The antibiotic treatment was followed by a two-week wash-out period on regular water. CD45.2^+^ BM cells (4x10^6^) either from BCG iv-vaccinated or *Mtb* iv-infected mice were intravenously injected into congenic CD45.1^+^ C57BL/6J mice 16 hours post lethal irradiation with 9 Gy. CD3^+^ T cells were depleted from the BM by MACS sorting prior to transplantation. Mice were kept under antibiotic treatment (0.5g Enrofloxacin (Bayer) per liter of drinking water) for three days before and two weeks after irradiation, along with anti-TB drugs for 5 days. The chimerism was validated after 16 weeks of reconstitution by flow cytometry and was > 96%. For second engraftment, CD45.2^+^ BM cells (4x10^6^) from 16 weeks reconstituted mice were intravenously injected into CD45.1^+^ C57BL/6J mice 16 hours post irradiation with 9 Gy. The chimerism was validated after 16 weeks of reconstitution by flow cytometry and was > 96% and subsequent experimentation was performed.

##### Generation of chimeric mice using inducible Tamoxifen model

CD45.1^+^ B6 mice were lethally irradiated with 9 Gy following 3 days of antibiotic treatment (0.5g Enrofloxacin (Bayer) per liter of drinking water). 16 hours later, the BM compartment was reconstituted with 4x10^6^ nucleated cells from Rosa26^Cre^ER^T2^Fth^lox/lox^ mice (CD45.2^+^) and antibiotic treatment was maintained for 2 additional weeks. Between 8-12 weeks post-injection, reconstitution was validated by flow cytometry and was > 90%. Tamoxifen was administered at 50mg/kg (10% ethanol in corn oil v/v) i.p. for 5 consecutive days and the mice were then rested for 1 week.

#### *In vitro* experiments

##### Generation of BMDM

BM from both femurs and tibiae was harvested in RPMI (Wisent) supplemented with 10% heat-inactivated FBS (Wisent). Cells were subsequently seeded in 7ml RPMI supplemented with 2mM L-glutamine, 10% FBS, 2% HEPES, 1% non-essential amino acids, 1% essential amino acids, 0.14% 5N NaOH, 1mM sodium pyruvate, 100U/ml penicillin, 100mg/ml streptomycin (all Wisent), 30% of L929-conditioned media (LCM) and isoniazid (INH 5μg/ml) in Petri dishes. After 3 days of incubation at 37°C with 5% CO2, fresh medium containing LCM and INH (5μg/ml) was added. Cells were allowed to differentiate into macrophages for a total of 6 days and then were harvested by removing the supernatant and addition of 4ml cell stripper (Corning) for 20 minutes at 37°C. As evaluated by flow cytometry, the purity was > 95%. INH was added only during the differentiation of the BMDM derived from BM cells of non-treated BCG-iv or *Mtb*-iv infected mice. Yield was determined by trypan blue dead cell exclusion counts and normalized to PBS control mice yields. For all experiments equal number of macrophages were seeded.

##### Macrophage Infection

BMDMs (1x10^6^ cells) were seeded in 6-well plates supplemented with RPMI without penicillin/streptomycin (1 ml) and incubated overnight at 37°C with 5% CO2. The next day, cells were infected with *Mtb* H37Rv (MOI 1), or BCG (MOI 10) unless otherwise indicated. Cells were incubated for 4 hours at 37°C with 5% CO2. Subsequently, cells were washed 3x with sterile PBS and were then incubated in supplemented RPMI without penicillin/streptomycin. CFUs were enumerated at 4 hours, day 3 and day 5 post-infection.

##### Mycobacterial CFU Enumeration

For CFU enumeration in *in vitro* infected cells, cells were lysed with 500μl sterile H_2_O for five minutes, followed by addition of 500μl PBS supplemented with 0.05% Tween80. For CFU enumeration in tissues, organs were homogenized in 1ml 7H9 broth (BD) supplemented with 0.2% glycerol (Wisent), 0.05% Tween80 (Fisher), and 10% ADC using OmniTip Plastic Homogenizer Probes (Omni International). Serial dilutions in PBS+0.05% Tween80 were plated on 7H10 agar plates with 10% OADC enrichment and PANTA (BD). Plates were then incubated at 37°C and counted after 21 days.

##### Western Blot

BM cells from BM *Fth*^*−/−*^ or BM *Fth*^*+/+*^ were isolated. Briefly, cells were lysed in lysis buffer (1% Triton X-100, 150mM NaCl, 20mM HEPES pH7.5, 10% glycerol, 1mM EDTA, supplemented with anti-protease and anti-phosphatase cocktails, Roche) and protein concentration was determined using BCA assay (Pierce). 20 μg of protein was resolved by SDS-PAGE and transferred onto PVDF membranes (Biorad). Membranes were blocked and incubated overnight at 4°C with gentle agitation with anti-Fth (1:1000, Cell Signaling Technologies) or actin (1:10 000, Sigma-Adrich). Then primary antibodies were conjugated to secondary HRP-conjugated antibodies and the signal was detected using Clarity ECL kit (Biorad) and acquired on Chemidoc MP System (Biorad).

##### Methylcellulose assay

5x10^3^ BM cells were suspended in methocult medium (MethoCult GF M3534; Stem Cell Technologies) with specific cytokines to promote the growth of myeloid progenitors. Colonies were counted after 10-12 days. Cells were collected by washing with PBS and stained for CD11b and cKit.

#### Quantification of IFN-I

Secretion of total active IFN-I (both IFN-α and IFN-β) in BM supernatants was assessed using B16-blue IFNα/β reporter cell line (InvivoGen), according to the specifications of the manufacturer.

#### Flow Cytometry

BM/Spleen cells (3 × 10^6^ cells) after RBC lysis were stained with fixable viability dye eFluor501 (eBioscience) at the concentration of 1:1000 for 30 minutes (4°C). Subsequently, the cells were washed with PBS supplemented with 0.5% BSA (Wisent) and incubated with anti-CD16/32 (clone 93, eBioscience) at a concentration of 1:100 in PBS/0.5% BSA at 4°C for 10 minutes except for myeloid progenitor and downstream progenitors staining. The following antibodies were then used for staining: anti-Ter-119 (clone Ter119), anti-CD11b (clone M1/70), anti-CD5 (clone 53-7.3), anti-CD4 (clone RM4-5), anti-CD8a (clone 53-6.7), anti-CD45R (clone RA3-6B2), and anti-Ly6G/C (clone RB6-8C5) all were biotin-conjugated (all BD Bioscience) and added at a concentration of 1:100 for 30 minutes at 4°C. Cells were subsequently washed with PBS/0.5% BSA. For staining of LKS, HSCs, and MPPs: Streptavidin–APC-Cy7 (eBioscience), anti-c-Kit–APC (clone 2B8, eBioscience), anti-Sca-1–PE-Cy7 (clone D7, eBioscience), anti-CD150–eFluor450 (clone mShad150, eBioscience), anti-CD48–PerCP-eFluor710 (clone HM48-1, BD Bioscience), anti-Flt3–PE (clone A2F10.1, BD Bioscience), and anti-CD34–FITC (clone RAM34, eBioscience) (all 1:100) were added and incubated at 4°C for 30 minutes. For staining of myeloid and lymphoid progenitors: Streptavidin–APC-Cy7 (eBioscience), anti-c-Kit–APC (clone 2B8, eBioscience), anti-Sca-1–PE-Cy7 (clone D7, eBioscience), anti-CD34–FITC (clone RAM34, eBioscience), anti-CD16/32 PerCP-eFluor710 (clone 93, eBioscience) and anti-CD127 BV786 (clone SB/199, BD bioscience) or anti CD127-BV605 (clone A7R34, Biolegend) (all 1:100) were added and incubated at 4°C for 30 minutes. For cMoPs and downstream progenitors: BM cells were incubated with biotin antibodies against lineage markers as mentioned above, except we included anti-Ly6G (clone 1A8-Ly6G) replacing anti-Ly6C/6G at 4°C for 30 minutes. Cells were subsequently washed with PBS/0.5% BSA. Following antibodies were added: Streptavidin–BUV-395(BD Bioscience), anti-c-Kit–Pacific Blue (clone 2B8, BD Bioscience), anti-Sca-1–PE-Cy7 (clone D7, eBioscience), anti-CD34–FITC (clone RAM34, eBioscience), anti-CD16/32–PerCP-efluor710 (clone 93, eBioscience), anti-CD115 BV711 (clone AFS98, BioLegend), anti-Flt3–PE (clone A2F10.1, BD Bioscience), anti-Ly6C–APC (clone HK1.4, eBioscience) and anti-Ly6G AF700 (clone 1A8–Ly6G, eBioscience) all (1:100 except Streptavidin BUV395 at 1:50) were added and incubated at 4°C for 30 minutes. In some experiments cells were further stained with AnnexinV–PE and 7AAD (Biolegend) or NucSpot Far-Red (Biotium), according to the manufacturer’s instructions and unfixed cells were acquired immediately. In another set of experiments, cells were fixed and permeabilized using the FOXP3 Transcription Factor Staining Kit (eBioscience) for 1 hour at 4°C. Then, cells were stained with anti-Ki67–PE (clone 16A8, BioLegend) (1:400) for 1 hour at 4°C and acquired.

##### Staining for innate and adaptive immune cells

Red blood cells were lysed in bone marrow and collagenase IV (Sigma)-treated lung samples. Lung, spleen or BM cells (3 × 10^6^) were then stained with fixable viability dye eFluor501 (eBioscience) at the concentration of 1:1 000 for 30 minutes (4°C). Subsequently, the cells were washed with PBS supplemented with 0.5% BSA (Wisent) and incubated with anti-CD16/32 (clone 93, eBioscience) at a concentration of 1:100 in PBS/0.5% BSA at 4°C for 10. After washing, cells were incubated with fluorochrome tagged antibodies at 4°C for 30 minutes. Antibodies for the innate panel: anti-CD11b–Pacific Blue (clone M1/70, eBioscience), anti-CD11c–PE-Cy7 (clone HL3, BD Bioscience), Siglec-F–PE-CF594 (clone E50–2440, BD Bioscience), F4/80–APC (clone BM8, eBioscience), Ly6C–FITC (clone AL-21, BD Bioscience), Ly6G–PerCP-eFluor710 (clone 1A8, eBioscience). Antibodies for the adaptive panel: anti-CD3–PE (clone 145-2C11, eBioscience), anti-CD19–PE-Cy7 (clone eBio1D3 (1D3), eBioscience), anti-CD4–eFluor450 or anti-CD4-FITC (clone GK1.5, eBioscience), anti-CD8–AF700 (clone 53-6.7, BD Bioscience). All cells were subsequently washed with PBS/0.5% BSA and resuspended in 1% paraformaldehyde.

##### Blood Leukocytes

50 μL of whole blood collected in heparin tubes (BD) was incubated with fluorochrome tagged antibodies at 4°C for 30 minutes. Antibodies for the innate panel: anti-CD11b–Pacific Blue (clone M1/70, eBioscience), anti-CD11c–PE-Cy7 (clone HL3, BD Bioscience), anti-Siglec-F–PE-CF594 (clone E50–2440, BD Biosciences), anti-F4/80–APC (clone BM8, eBioscience), anti-Ly6C–FITC (clone AL-21, BD Bioscience), anti-Ly6G–PerCP–eFluor710 (clone 1A8, eBioscience). Antibodies for the adaptive panel: anti-CD3–PE (clone145-2C11, eBioscience), anti-CD19–PE-Cy7 (clone eBio1D3 (1D3), eBioscience), anti-CD4–FITC (clone GK1.5, eBioscience), anti-CD8 AF700 (clone 53-6.7, BD Bioscience). After RBC lysis, cells were subsequently washed with PBS/0.5% BSA and resuspended in 1% paraformaldehyde.

If required, panels were modified to contain anti-CD45.1–APC (clone A20, BD Bioscience, 1:100) and anti-CD45.2–BUV395 (clone 104, BD Bioscience, 1:100).

Cells were acquired on the Fortessa-X20 (BD) and analyzed using FlowJo software (version 10.6.1). All percentages are of single viable frequency, unless otherwise indicated.

##### Evaluation of Mitochondrial Fe^2+^, Mitotracker Green/Orange and Mitosox Red

BM mononuclear cells were stained with rhodamine B 4-[(1,10-phenanthrolin-5-yl)aminocarbonyl]benzyl ester (RPA) (Squarix) at 0.2 μM, or Mitotracker Green and Orange 150n5M, or MitoSox Red 1μM (all from Invitrogen technologies) in PBS for 30 min at room temperature and then washed with PBS. The dye-loaded cells were further stained with antibodies for progenitors as mentioned above. RPA MFI fluorescence was normalized to PBS groups, inversed and multiplied by 100 to give a percentage of iron pools compared to PBS, as fluorescence is quenched by higher levels of iron. For experiments involving mitochondrial potential, dysregulated mitochondria were considered as Mitotracker Green^hi^ and Mitotracker Orange^lo^ as previously described (Ip et al., 2017).

#### ImageStream

Freshly isolated BM cells from naive C57BL/6J mice were infected with *Mtb*-GFP or BCG-GFP (MOI 3) for 4 hours. BM cells were then stained with antibodies for LKS and Lineage^+^ cells. Images were captured on an Amnis ImageStream Mark II Imaging Flow Cytometer with 40X magnification (EMD Millipore). Data were acquired and analyzed using Amnis INSPIRE software and Amnis IDEAS software, respectively. ImageStream samples were also acquired using Fortessa-X20 (BD) and then analyzed using FlowJo software.

#### Cell Sorting

For stem cell sorting, BM cells were harvested from femurs, tibiae, humeri and ilia, and incubated with the described biotin-conjugated antibodies against lineage-committed cells. Subsequently, the cells were incubated with MACS streptavidin conjugated microbeads (Miltenyi) for 30 minutes at 4°C. A depletion of the lineage-committed cells was performed using MACS magnets with LS Columns (Miltenyi, #130-042-401). The remaining cells were incubated with the above described antibodies for LKS/HSCs/MPPs. DAPI (0.005μg) was added for viability staining to 10x10^6^ cells right before sorting on a BD FACSAria Fusion sorter. Cells were sorted in 200μl lysis buffer of Magmax-96 Total RNA Isolation Kit (life technologies, Ambion 1830) at room temperature or in PBS containing 0.04% BSA for single cell sequencing. RNA samples were vortexed for 30 s, and subsequently stored at –80°C until RNA extraction.

#### Perls’ Prussian blue iron staining

Bone epiphyses were removed and femurs were fixed in 4% PFA for 24-48h followed by washing with PBS. Perls’ staining was performed by Histopathology Core of RI-MUHC, Montreal, QC, Canada.

#### Bulk RNA-seq library preparation and sequencing

Total RNA was extracted from the sorted HSCs and MPPs using Magmax-96 Total RNA isolation kit (life technologies, Ambion 1830M). RNA quality was evaluated spectrophotometrically, and quality was assessed with the Agilent 2100 Bioanalyzer (Agilent Technologies). All samples showed RNA integrity number > 8. RNA-sequencing libraries were prepared using the SMARTer Stranded RNA-Seq Kit (Clontech). Once prepared, indexed cDNA libraries were pooled in equimolar amounts and were sequenced with single-end 100 bp reads on an Illumina HiSeq2500.

#### Single cell RNA-seq library preparation and sequencing

Single cell transcriptomic data was collected for LKS (Lin^-^ Sca-1^+^ cKit^+^) and myeloid progenitor (Lin^-^ Sca-1^-^ cKit^+^) populations in the context of the *Mtb* aerosol challenge and the second engraftment experiments. Cells were sorted as described above into PBS containing 0.04% BSA.

Single-cell GEMs were generated using a Chromium Controller instrument (10x Genomics). Sequencing libraries were prepared using Chromium Single Cell 3′ Reagent Kits (10x Genomics), according to the manufacturer’s instructions. Briefly, GEM-RT was performed in a thermal cycler: 53°C for 45 min, 85°C for 5 min. cDNA was cleaned up with DynaBeads MyOne Silane Beads (ThermoFisher Scientific) and amplified with a thermal cycler: 98°C for 3 min, cycled 12 × 98°C for 15 s, 67°C for 20 s, 72°C for 1 min, and 72°C 1 min. After a cleanup with SPRIselect Reagent Kit, the libraries were constructed by performing the following steps: fragmentation, end-repair, A-tailing, SPRIselect cleanup, adaptor ligation, SPRIselect cleanup, sample index PCR, and SPRIselect size selection. Libraries were sequenced on a NovaSeq S2 flowcell with 100 bp paired-end reads.

### Quantification and Statistical Analyses

#### Statistics for mouse work

Statistical analyses were performed using Graph Pad Prism, version 8. Data are displayed as mean ± SEM. Each symbol represents an individual mouse, or an individual n value is listed in the figure legends. Statistical significance was determined by t test, one-way ANOVA, two-way ANOVA, or log-rank test as indicated in the figure legends, and significance is represented by the following scheme: ^∗^p < 0.05, ^∗∗^p < 0.01, ^∗∗∗^p < 0.001, ^∗∗∗∗^p < 0.0001.

#### Bulk RNA-seq

##### RNA-seq Analyses: Pre-processing Steps

Experimental mice were split into three treatment groups, (either BCG-iv, *Mtb*-iv or the control: PBS-iv) with 4 animals each. 28 days after treatment, animals were sacrificed, and their BM extracted. Hematopoietic stem cells (HSCs; Lin^-^ cKit^+^ Sca-1^+^ CD150^+^) and multipotent progenitors (MPPs; Lin^-^ cKit^+^ Sca-1^+^ CD150^-^) were sorted as described above. RNA was then extracted and sequenced, generating a dataset of 23 samples (two cell types multiplied by three treatments and by four animals per group, minus one HSC-PBS sample that did not produce a valid RNA library).

As for RNA-seq data pre-processing, low-quality score bases (Phred score < 20) and adaptor sequences were trimmed using Trim Galore (version 0.2.7) (http://www.bioinformatics.babraham.ac.uk/projects/trim_galore/). Mapping of the resulting reads to the mouse genome reference sequence (Ensembl GRCm38 release 81) was done with kallisto (v0.43.0) (Bray et al., 2016). Then, protein coding genes were selected, and samples were normalized using the weighted trimmed mean of M-values algorithm (TMM), implemented in edgeR (Robinson et al., 2010). Data were log-transformed using voom, within the limma package (Ritchie et al., 2015) and lowly-expressed genes were filtered out, defined as those with a median log2-transformed expression lower than 2 within all experimental groups (i.e., treatment-cell-type combinations). This produced a reads matrix of 12132 genes.

##### Differential expression analyses

The filtered matrix was then re-normalized, using EdgeR and voom, and modeled according to the following design, using limma (Ritchie et al., 2015):

Expression∼Cell_type+Treatment:cell_type

From the mentioned model design run over all 12132 genes in the dataset, BCG and *Mtb* effects were retrieved within each cell type (Table S1, Tabs i-iv). Focus was then put on genes showing treatment responses in any cell-type (2214 genes responding at 1% FDR to either BCG or *Mtb* at HSCs; 3542 for MPPs) and these genes were then tested for differences in expression between the two treatments (Table S1, tabs v-vi). Differences in expression between HSCs and MPPs were also characterized (Table S1, tabs vii-ix). We found 581, 608 and 600 differentially expressed genes between the HSCs and MPPs, at 1% FDR, in cells coming from PBS, BCG and *Mtb* treated mice, respectively.

#### scRNA-seq

##### scRNA-seq data pre-processing and quality control

Sample preprocessing was done using the count command of CellRanger (Zheng et al., 2017) against the mm10 1.2.0 reference genome, yielding numbers of cells between 3,305 and 11,994 depending on experiment, cell population and condition (Table 1, row 1). Initially, we measured the expression of 31053 genes in each cell (Table 1, row 2). In each of the four datasets analyzed, we excluded lowly expressed genes, detected in less than 5 cells in all conditions, in order to select a relevant gene set in each experiment to conduct quality control on (Table 1, row 3). Using this reference gene set in each experiment, we determined the percentage of protein coding genes detected, as well as the percentage of mitochondrial RNA in each cell. We then excluded cells that featured: *(i)* a high fraction of mitochondrial RNA reads ($f_{mito}>5\%$), *(ii)* a low percentage of protein-coding genes ($f_{pc}<95\%$) or (iii) a number of UMIs that was either too low (< $n_{UMI}<5000$) or too high ($n_{UMI}>40,000$). After the filtering, we kept variable numbers of cells per experiment and condition, comprised between 578 and 4733 (Table 1, row 4). Using this final set of cells, we filtered out genes detected in less than five cells in all conditions and re-normalized the percentages of protein coding genes and mitochondrial transcripts per cell on the final dataset. At the end, the number of genes included for clustering and differential expression analyses varied between 14568 and 16480, depending on the experiment and cell population (Table 1, row 5).

**Table 1:** Recapitulation of Number of Cells and Genes at the Different Stages of the Quality Control Process of the scRNA-Seq Data Analyses

	*Mtb.* Aerosol Challenge				Serial Engraftment					
	LKS		Progenitors		LKS			Progenitors		
	PBS	*Mtb*	PBS	*Mtb*	PBS	BCG	*Mtb*	PBS	BCG	*Mtb*
Cells	3,305	6,580	3,741	8,259	5,391	4,673	2,786	5,094	8,987	11,994
Genes	31,053		31,053		31,053			31,053		
Genes > 5 cells	16,071		15,103		17,019			16,268		
Filtered cells	2,388	3,310	2,241	3,504	3,872	2,524	1,563	3,353	5,020	1,486
Filtered genes	15,550		14,568		16,480			15,676		

##### scRNA-seq data normalization

We used the function computeSumFactors, from the R package scran (Lun et al., 2016) to normalize the data. This method works by declaring a series of cell pools whose expressions are added and normalized against a common reference. Each of these pools is then interpreted as a linear system from which the contribution of each single cell can be deconvolved, allowing the inference of cellwise normalization factors in a way that is robust against noise and low expression levels. To ensure that a majority of genes are not DE between any of the pools and the common reference that they are normalized against, clusters of homogeneous gene expression are first built using the function quickCluster. Thus, cell specific normalization factors $n_{i}$ are inferred within each cluster, and then rescaled by normalization between clusters. Normalization used was:

(1) $$E_{norm}\left(i,j\right)=log_{2}(1+E_{count}\left(i,j\right)/n_{i})$$

For the j-th gene in the i-th cell, where $n_{i}$ represent the normalizatiomn factor estimated for the i-th cell.

##### Correcting for variation in cell cycle in single cell data

Cell cycle numeric scores were inferred for each cell using the function cyclone implemented in package scran (Lun et al., 2016). The method is based on a pre-defined classifier constituted by gene pairs whose relative difference in expression depends on the cell cycle stage. By comparing the coherence of the sign of the differences observed in the data to the classifier’s expectation, per-cell scores were obtained for phases G1 and G2M. These values were summarized as a single numeric score C = (G1-G2M)/(G1+G2M), which we mean centered and scaled across cells. These scores were corrected for at the steps of expression data scaling and cell clustering.

##### Single cell data scaling

Once the data was normalized in each of the four datasets, we fitted for each gene the following linear model:

(2) $$E∼f_{pc}+f_{mito}+n_{UMI}+C+\varepsilon$$

The variance associated to the residuals ε derived from this model $s_{\varepsilon }^{2}$ was then fitted against mean expression of the corresponding genes using scran’s smoothing function trendVar (parameters: method = loess, span = 0.1, degree = 2). The deviations from the inferred trend were computed per gene using scran’s function decomposeVar. Then, the first decile corresponding to the genes showing the largest, negative deviations from the fitted trend were selected, and the procedure was repeated. By doing so, we obtained an unbiased basal trend for the technical component of the residual variance that is not affected by the outliers with variance much larger than the technical variance. Finally, we calculated the biological component of the variance as the difference between the residual variance and the basal technical component:

(3) $$s_{\varepsilon }^{2}=s_{tech}^{2}+s_{bio}^{2}$$

Once the variance decomposition is ready, the scaled expression $\tilde{E}$ was defined by rescaling the residuals ε of Equation (2) by the square-root of the technical component of the residual variance, from Equation (3)

(4) $$\tilde{E}=\varepsilon /s_{tech}$$

By doing this, the mean-variance relationship was successfully removed from the data.

##### Selection of highly informative genes

Next, we integrated the data corresponding to the different conditions. To do so, we first selected in each case a set of highly informative genes (HIGs) whose expression is to be interrogated to complete data integration. In both LKS^+^ and progenitor datasets, we selected HIGs as those that showed both high levels of (biological) expression variance across cells and had been reported in the literature as markers of the different cell sub-populations present among LKS^+^ and progenitor cells (derived from Cabezas-Wallscheid et al., 2014, for LKS^+^ data and Paul et al., 2015, for progenitor cells). Specifically, for LKS^+^ cells, HIGs were defined as the intersection between the following four sets of genes: (set i) the 1000 genes with mean expression higher than 0.1 that showed the largest positive biological variance $s_{bio}^{2}$ across cells in our data (‘‘highly variable genes’’. HVGs); (set ii) genes differently expressed (10% FDR) between MPPs and HSCs based on the bulk RNA-seq, in all conditions present in each experiment (Table S1, tabs vii-ix, 412 genes), (set iii) genes differently expressed between myeloid progenitors (MPP3) and lymphoid progenitors (MPP4) (219 genes) based on the bulk RNA-seq data reported in Cabezas-Wallscheid et al. (2014) and (set iv) Genes differentially expressed between short and long term hematopoietic stem cells (105 genes) based on the bulk RNA-seq data also reported in Cabezas-Wallscheid et al. (2014). Regarding set (iv), it is important to note that the gating strategy followed in Cabezas-Wallscheid et al. (2014) is slightly different from ours. While we do not distinguish among sub-types of long-term HSCs (according to our gating strategy, LT-HSCs are just Flt3^-^, CD48^-^ and CD150^+^), Cabezas-Wallscheid et al. (2014) subdivide this population according to CD34 levels, distinguishing between cells that are Flt3^-^, Cd48^-^, CD150^+^, CD34^-^ (that they denote as *HSCs*), and cells that are Flt3^-^,CD48^-^, CD150^+^, CD34^+^ (that they denote as *MPP1*s). In what regards short term HSCs, the cell population that they denote MPP2s (Flt3^-^, CD48^+^ and CD150^+^ and CD34^+^) would be included within the population described as short-term HSCs in this work (Flt3^-^, CD48^+^ and CD150^+^). Consequently, in order to obtain genes that would mark the difference between LT- and ST-HSCs according to our gating schematics (set iv), we selected the genes that, at the same time, showed differential expression between HSCs and MPP2 and MPP1 versus MPP2 in the dataset from Cabezas-Wallscheid et al. (2014) according to their notation.

In what regards the identification of highly informative genes to identify sub-populations in the progenitor cells data, we used the single cell data published in Paul et al. (2015) where an analogous population of hematopoietic progenitors (c-Kit^+^ Sca1^-^ Lin^-^) was sorted and analyzed using sc-RNaseq. From the dataset reported in this work, we extracted cluster specific fold changes for the genes showing statistically significant associations to any of the cell clusters reported in the study, and kept those that present, for some of the clusters, a fold change larger than 1.5 with respect to the average expression levels across the rest of cells in the dataset. In this case, HIGs were defined as the intersection of these genes and HVGs in our data. Following these procedures, we selected 349, 115 HIG in the aerosol LKS and progenitor cells, respectively, and 346 and 112 HIGs in LKS and progenitor cells, respectively, in the secondary engraftment dataset.

##### Data integration across conditions

Data were integrated across conditions using the anchoring routine proposed in Seurat (FindIntegrationAnchors) (Satija et al., 2015; Stuart et al., 2019), applied to the sub-matrix constituted by the highly informative genes previously identified. This technique works by identifying pairs of cells across conditions that represent common cell states (anchors), despite batch/condition effects, that are then used to provide a transformed (integrated) expression matrix $E_{int}$ where anchors will ultimately be assigned to common cell clusters. The procedure has the advantage of allowing integration over multiple samples at the same time (useful in the engraftment experiment, where we had to integrate the data across three conditions (PBS, BCG-iv and *Mtb*-iv), and features flexibility in the assignment of anchors, allowing for the possibility that some cell types are unique to one of the conditions. After data integration, the steps of variance modeling, scaling and identification of highly informative genes were repeated on the transformed data, with a procedure entirely analogous to what was previously described.

##### Cell clustering

The scaled version of the integrated data was used to identify different cell sub-populations in each of the datasets available. First, we performed a principal component analysis of the matrix of scaled & integrated expression levels of the HIGs identified from the integrated data. After visual inspection of elbow plots, we select the first n = 20 PCs (with the exception of LKS cells in the engraftment experiment for which we selected n = 15) to identify cell clusters using Seurat’s functions FindNeighbors plus FindClusters. We used the default parameters except for the resolution, which was set at 0.4 for the engraftment experiment and aerosol LKS^+^ cells, 0.7 for the aerosol progenitors. In parallel, we run Uniform Manifold Approximation and Projection (UMAP) to visualize the clustering using RunUMAP, also from Seurat.

##### Cluster classification and validation of sub-populations (LKS)

To distinguish hematopoietic stem cells (HSC), from multipotent progenitors (MPP), we used the genes differentially expressed between sorted HSCs and MPPs in the bulk RNA-seq experiments reported in Table S1, tabs vii-ix (set ii, 412 genes). We stratified this gene-set according to the direction of the effects and computed in each cell the average integrated scaled expression of all genes that were respectively up or downregulated in HSCs versus MPPs. By comparing the expression levels of these averaged cell-type markers across clusters we could classify HSCs and MPPs in each experiment. This allowed the identification of 4 clusters of HSCs and 2 clusters of MPPs in the aerosol experiment, and 4 HSC and 3 MPP clusters in the second engraftment experiment. (Figure S3, Figure S7O and S7V, upper panels). Next, to distinguish between myeloid-biased (MPP3) and lymphoid-biased (MPP4) progenitors, we used the set of 219 genes (set iii) that were reported in Cabezas-Wallscheid et al. (2014) to be differentially expressed between these two populations according to the direction of effects, and computed the average expression values of these genes (integrated and scaled) in each cell. Focusing on the clusters previously defined as MPPs, we visualized the expression of these average markers of lymphoid versus myeloid up or downregulation to distinguish in each experiment, clusters of MPP3 versus MPP4 cells (Figure S3, Figure S7Q and S7X, center). While in the aerosol experiment we observed one cluster associated to each phenotype, in the second engraftment we identified two clusters of MPP3s and one cluster of MPP4s. Finally, in order to distinguish short versus long term HSCs, we built analogous average markers from the set of genes differentially expressed between these two cell sub-types as defined by Cabezas-Wallscheid et al. (2014). Exploring the distribution of these average markers across HSC clusters led to the identification of a marked distinction between one LT- versus three ST-HSCs in the aerosol experiment, and a more gradual distribution of transcriptional profiles, which can be associated to the existence of two versus two clusters of LT- versus ST-HSCs in the second engraftment dataset (Figure S3, Figure S7O and S7V, bottom)

##### Cluster classification and validation of sub-populations (progenitor populations)

In order to identify subpopulations of myeloid progenitors associated to the different developmental stages and lineage biases present in the cell population initially sorted (c-Kit^+^ Sca1^-^ Lin^-^), we capitalized in the data published in Paul et al. (2015), where the same cell population was analyzed through scRNA-seq. In that work, the authors identified 19 clusters that they associate to either Common myeloid progenitors (CMPs), Granulocyte-Monocyte progenitors (GMPs) or Megakaryocyte-erythrocyte progenitors (MEPs). Within each of these broader groups, they further identified subpopulations that were polarized toward specific cell fates: CMPs biased toward a megakaryocytic cell fate, groups of MEPs distributed in a continuous gradient of bias toward erythrocyte differentiation, as well as CMPs and GMPs showing bias toward different myeloid cells: monocytes, neutrophils, eosinophils and basophils. As part of their analyses, they report a set of 3,461 genes that are specific markers of each of these clusters, providing an expression estimate of the gene in each cluster, measured as the fold change of the observed expression of a given gene, in a given cluster, compared to its average expression across all clusters.

In order to compare these data to our clustering results, we selected the subset of their cluster markers that were among the set of HIGs in the integrated data. Then, we retrieved, for each of these genes, cluster-specific expression estimates by fitting a linear model

(5) $${\tilde{E}}_{int}∼0+Cluster$$

Which produced a cluster specific intercept $\beta_{o}\left(i,j\right)$, for the ith gene under scrutiny, in the jth cluster. From these cluster-specific intercepts, (which operate in log-scale) we reversed the log-transformation to retrieve a natural scale expression estimate for the expression of gene I in cluster j as $2^{\beta_{o}\left(i,j\right)}$, which we averaged across clusters for each gene: ${\left\langle 2^{\beta_{o}\left(i,j\right)}\right\rangle }_{j}$. Finally, by obtaining the ratio between those two quantities, we retrieved an estimate of the expression fold change of any given cluster with respect to the average of all clusters.

(6) $$FC\left(i,j\right)=2^{\beta_{o}\left(i,j\right)}/{\left\langle 2^{\beta_{o}\left(i,j\right)}\right\rangle }_{j}$$

These values are equivalent to the fold changes reported in Paul et al. (2015) and were used to identify matches between our cell clusters and those reported by Paul et al. (2015) based on the correlation structure of the two datasets (Figure S3, Figure S7P and S7W). Cell clustering assignment was further validated by inspecting the expression levels of canonical lineage markers (Figure S3, Figure S7Q and S7X). MEP expressed Car1, Car2, Klf1 high; CD34, Cebpa, and Spi1 low; GMPs expressed Car1, Car2, Klf1 low, CD34, Cebpa, Spi1 high; and CMPs expressed Meis1 high while GMP and MEP markers were lowly expressed.

Within MEP cells, we further analyzed a series of markers that were reported to either increase or decrease continuously in earlier or more mature erythrocyte progenitors (Car1, Car2, Cited4, Epor, Gfi1b, Klf1, and Phf10; whose expression increases in more mature cells, and Gata2 and Meis1, which decreases). As it was observed in Paul et al. (2015) our clusters of erythroid progenitors can be mapped to different developmental stages, from MEP_EP1 (earlier) to MEP_EP3 (more mature).

Within GMPs, neutrophil progenitors (GMP_NPs) were characterized by high expression levels of neutrophilic markers Cebpe and Gfi1; basophil progenitors (GMP_BPs) by high expression of basophile marker Lmo4; and Monocyte progenitors (GMP_MonP) by high amounts of Irf8.

##### Within-cluster differential gene expression induced by BCG and Mtb

Finally, we interrogated for differences in expression of genes in response to BCG or *Mtb* challenges. Since one of the putative effects of BCG and/or *Mtb* on cell phenotypes might be the promotion or arrest of cell quiescence, we used a linear model where cell cycle is not corrected for, in order to avoid masking the putative effects of treatment on the expression patterns of cell cycle-dependent genes. It is also important to note, that to avoid the distortion induced in the data by the procedures of data scaling and integration, to conduct differential expression analyses, we used the normalized, original data prior to these procedures used in cell clustering. To do these analyses, we fitted the following experimental design:

(7) $$E∼f_{pc}+f_{mito}+n_{UMI}+Cluster+Condition:Cluster+\varepsilon$$

The results of these analyses, for each cluster, are reported in Tables S4, S5, and S6 and summarized in Figures 3 and 7, evidencing the existence of relevant effects within each of the cell clusters identified, in the four datasets here analyzed.

#### GO enrichments and visualization

Gene ontology (GO) enrichment analyses were performed using the Cytoscape module ClueGO (Bindea et al., 2009; Shannon et al., 2003). We conducted one-sided tests for enrichment and corrected for multiple tests using the Benjamini-Hochberg (B-H) method (Benjamini and Hochberg, 1995). For visualization, in Figure S2B and the Table S2 (bulk RNA-seq data) and Table S7 (serial engraftment experiment, sc-RNA-seq data), analyses were focused only on terms that fell between levels 4 and 7 of the GO tree in the (Biological Process). In all analyses, we included terms that had at least 10 genes in the test gene-set and for which at least 20% of the total number of genes belonging to the GO term were present in the test gene set, except in the case of bulk-RNA-seq MPPs, where thresholds were raised to a minimum of 15, and 35%. Finally, to compile Figure S2A, which shows the existing correlation between GO-enrichment results after BCG versus *Mtb* treatments in each cell type, analyses were repeated removing the thresholds on gene-set size or percentage in order to avoid any selection bias. In this analysis we included all terms between levels 4 and 7 of the GO tree, regardless of enrichment significance or gene-set size. Importantly, when done, a much larger number of GO terms passed the enrichment significance threshold (between 1.4 and 7.1 times more terms enriched at 5% FDR, depending on the combination of cell-type and treatment), despite the much larger set of terms tested.

Finally, GSEA (https://www.gsea-msigdb.org/gsea/) was used to interrogate functional enrichments among genes showing differences in expression between BCG and *Mtb* treated mice, in each cell type (Figure 2D; Table S3). It was again used to interrogate for functional enrichments among genes differentially expressed upon treatment, either *Mtb* alone in the aerosol challenge experiment Figure 3S), or both BCG and *Mtb* in the serial engraftment section (Figures 7T and 7U). In all cases, we used the hallmark list of genesets, and conducted GSEA on ranks of standardized differences in expression, (mode GSEA pre-ranked, version 6.2).

## Supplemental Information

Table S1. Bulk RNA-Seq Differential Gene Expression in HSCs and MPPs between *Mtb* and BCG, Related to Figure 2

Table S2. Bulk RNA-Seq Gene Ontology Enrichment Results for *Mtb* versus BCG in MPPs and HSCs, Related to Figure 2

Table S3. Bulk RNA-Seq GSEA Results for MPPs and HSCs of BCG versus *Mtb*, Related to Figure 2

Table S4. DEGs following scRNA-Seq of Mice that Received Aerosol Treatment, Related to Figure 3

Table S5. DEGs following scRNA-Seq of Secondary Engraftment Mice in *Mtb* versus PBS, Related to Figure 7

Table S6. DEGs following scRNA-Seq of Secondary Engraftment Mice in BCG versus PBS, Related to Figure 7

Table S7. scRNA-Seq Gene Ontology Enrichment Analyses following Secondary Engraftment, Related to Figure 7

## References

[bib1] Akashi K., Kondo M., von Freeden-Jeffry U., Murray R., Weissman I.L. (1997). Bcl-2 rescues T lymphopoiesis in interleukin-7 receptor-deficient mice. Cell.

[bib2] al-Rafaie F.N., Wilkes S., Wonke B., Hoffbrand A.V. (1994). The effect of deferiprone (L1) and desferrioxamine on myelopoiesis using a liquid culture system. Br. J. Haematol..

[bib3] Altare F., Durandy A., Lammas D., Emile J.F., Lamhamedi S., Le Deist F., Drysdale P., Jouanguy E., Doffinger R., Bernaudin F. (1998). Impairment of mycobacterial immunity in human interleukin-12 receptor deficiency. Science.

[bib4] Alter A., de Leseleuc L., Van Thuc N., Thai V.H., Huong N.T., Ba N.N., Cardoso C.C., Grant A.V., Abel L., Moraes M.O. (2010). Genetic and functional analysis of common MRC1 exon 7 polymorphisms in leprosy susceptibility. Hum. Genet..

[bib5] Antonelli L.R., Gigliotti Rothfuchs A., Goncalves R., Roffe E., Cheever A.W., Bafica A., Salazar A.M., Feng C.G., Sher A. (2010). Intranasal Poly-IC treatment exacerbates tuberculosis in mice through the pulmonary recruitment of a pathogen-permissive monocyte/macrophage population. J. Clin. Invest..

[bib6] Baldridge M.T., King K.Y., Boles N.C., Weksberg D.C., Goodell M.A. (2010). Quiescent haematopoietic stem cells are activated by IFN-gamma in response to chronic infection. Nature.

[bib7] Barber D.L., Mayer-Barber K.D., Feng C.G., Sharpe A.H., Sher A. (2011). CD4 T cells promote rather than control tuberculosis in the absence of PD-1-mediated inhibition. J. Immunol..

[bib8] Behar S.M., Divangahi M., Remold H.G. (2010). Evasion of innate immunity by Mycobacterium tuberculosis: is death an exit strategy?. Nat. Rev. Microbiol..

[bib9] Bekkering S., Quintin J., Joosten L.A., van der Meer J.W., Netea M.G., Riksen N.P. (2014). Oxidized low-density lipoprotein induces long-term proinflammatory cytokine production and foam cell formation via epigenetic reprogramming of monocytes. Arterioscler. Thromb. Vasc. Biol..

[bib10] Belyaev N.N., Brown D.E., Diaz A.I., Rae A., Jarra W., Thompson J., Langhorne J., Potocnik A.J. (2010). Induction of an IL7-R(+)c-Kit(hi) myelolymphoid progenitor critically dependent on IFN-gamma signaling during acute malaria. Nat. Immunol..

[bib11] Benjamini Y., Hochberg Y. (1995). Controlling the False Discovery Rate - a Practical and Powerful Approach to Multiple Testing. J. R. Stat. Soc. B.

[bib12] Bindea G., Mlecnik B., Hackl H., Charoentong P., Tosolini M., Kirilovsky A., Fridman W.H., Pages F., Trajanoski Z., Galon J. (2009). ClueGO: a Cytoscape plug-in to decipher functionally grouped gene ontology and pathway annotation networks. Bioinformatics.

[bib13] Blankenhaus B., Braza F., Martins R., Bastos-Amador P., Gonzalez-Garcia I., Carlos A.R., Mahu I., Faisca P., Nunes J.M., Ventura P. (2019). Ferritin regulates organismal energy balance and thermogenesis. Mol. Metab..

[bib14] Bray N.L., Pimentel H., Melsted P., Pachter L. (2016). Near-optimal probabilistic RNA-seq quantification. Nat. Biotechnol..

[bib15] Bronte V., Pittet M.J. (2013). The spleen in local and systemic regulation of immunity. Immunity.

[bib16] Cabezas-Wallscheid N., Klimmeck D., Hansson J., Lipka D.B., Reyes A., Wang Q., Weichenhan D., Lier A., von Paleske L., Renders S. (2014). Identification of regulatory networks in HSCs and their immediate progeny via integrated proteome, transcriptome, and DNA methylome analysis. Cell Stem Cell.

[bib17] Chackerian A.A., Alt J.M., Perera T.V., Dascher C.C., Behar S.M. (2002). Dissemination of Mycobacterium tuberculosis is influenced by host factors and precedes the initiation of T-cell immunity. Infect. Immun..

[bib18] Cirovic B., de Bree L.C.J., Groh L., Blok B.A., Chan J., van der Velden W., Bremmers M.E.J., van Crevel R., Handler K., Picelli S. (2020). BCG Vaccination in Humans Elicits Trained Immunity via the Hematopoietic Progenitor Compartment. Cell Host Microbe.

[bib19] Comas I., Chakravartti J., Small P.M., Galagan J., Niemann S., Kremer K., Ernst J.D., Gagneux S. (2010). Human T cell epitopes of Mycobacterium tuberculosis are evolutionarily hyperconserved. Nat. Genet..

[bib20] Das B., Kashino S.S., Pulu I., Kalita D., Swami V., Yeger H., Felsher D.W., Campos-Neto A. (2013). CD271(+) bone marrow mesenchymal stem cells may provide a niche for dormant Mycobacterium tuberculosis. Sci. Transl. Med..

[bib21] de Bruin A.M., Demirel O., Hooibrink B., Brandts C.H., Nolte M.A. (2013). Interferon-gamma impairs proliferation of hematopoietic stem cells in mice. Blood.

[bib91] Divangahi M., Desjardins D., Nunes-Alves C., Remold H.G., Behar S.M. (2010). Eicosanoid pathways regulate adaptive immunity to Mycobacterium tuberculosis. Nat Immunol..

[bib22] Divangahi M., Mostowy S., Coulombe F., Kozak R., Guillot L., Veyrier F., Kobayashi K.S., Flavell R.A., Gros P., Behr M.A. (2008). NOD2-Deficient Mice Have Impaired Resistance to Mycobacterium tuberculosis Infection through Defective Innate and Adaptive Immunity. J. Immunol..

[bib23] Divangahi M., Chen M., Gan H., Desjardins D., Hickman T.T., Lee D.M., Fortune S., Behar S.M., Remold H.G. (2009). Mycobacterium tuberculosis evades macrophage defenses by inhibiting plasma membrane repair. Nat. Immunol..

[bib24] Dolznig H., Habermann B., Stangl K., Deiner E.M., Moriggl R., Beug H., Mullner E.W. (2002). Apoptosis protection by the Epo target Bcl-X(L) allows factor-independent differentiation of primary erythroblasts. Curr. Biol..

[bib25] Essers M.A., Offner S., Blanco-Bose W.E., Waibler Z., Kalinke U., Duchosal M.A., Trumpp A. (2009). IFNalpha activates dormant haematopoietic stem cells in vivo. Nature.

[bib26] Gangaidzo I.T., Moyo V.M., Mvundura E., Aggrey G., Murphree N.L., Khumalo H., Saungweme T., Kasvosve I., Gomo Z.A., Rouault T. (2001). Association of pulmonary tuberculosis with increased dietary iron. J. Infect. Dis..

[bib27] Ganz T. (2013). Systemic iron homeostasis. Physiol. Rev..

[bib28] Genewein A., Telenti A., Bernasconi C., Mordasini C., Weiss S., Maurer A.M., Rieder H.L., Schopfer K., Bodmer T. (1993). Molecular approach to identifying route of transmission of tuberculosis in the community. Lancet.

[bib29] Gozzelino R., Soares M.P. (2014). Coupling heme and iron metabolism via ferritin H chain. Antioxid. Redox Signal..

[bib30] Hartner J.C., Walkley C.R., Lu J., Orkin S.H. (2009). ADAR1 is essential for the maintenance of hematopoiesis and suppression of interferon signaling. Nat. Immunol..

[bib31] Hood M.I., Skaar E.P. (2012). Nutritional immunity: transition metals at the pathogen–host interface. Nat. Rev. Microbiol..

[bib32] Hunt B.J., Andrews V., Pettingale K.W. (1987). The significance of pancytopenia in miliary tuberculosis. Postgrad. Med. J..

[bib33] Ioannou S., Hatzis G., Vlahadami I., Voulgarelis M. (2010). Aplastic anemia associated with interferon alpha 2a in a patient with chronic hepatitis C virus infection: a case report. J. Med. Case Reports.

[bib34] Ip W.K.E., Hoshi N., Shouval D.S., Snapper S., Medzhitov R. (2017). Anti-inflammatory effect of IL-10 mediated by metabolic reprogramming of macrophages. Science.

[bib35] Kamada R., Yang W., Zhang Y., Patel M.C., Yang Y., Ouda R., Dey A., Wakabayashi Y., Sakaguchi K., Fujita T. (2018). Interferon stimulation creates chromatin marks and establishes transcriptional memory. Proc. Natl. Acad. Sci. USA.

[bib36] Kanayama M., Xu S., Danzaki K., Gibson J.R., Inoue M., Gregory S.G., Shinohara M.L. (2017). Skewing of the population balance of lymphoid and myeloid cells by secreted and intracellular osteopontin. Nat. Immunol..

[bib37] Kaufmann E., Sanz J., Dunn J.L., Khan N., Mendonca L.E., Pacis A., Tzelepis F., Pernet E., Dumaine A., Grenier J.C. (2018). BCG Educates Hematopoietic Stem Cells to Generate Protective Innate Immunity against Tuberculosis. Cell.

[bib38] Kee B.L. (2011). A comprehensive transcriptional landscape of human hematopoiesis. Cell Stem Cell.

[bib39] Kondo M., Weissman I.L., Akashi K. (1997). Identification of clonogenic common lymphoid progenitors in mouse bone marrow. Cell.

[bib40] Lai A.Y., Kondo M. (2006). Asymmetrical lymphoid and myeloid lineage commitment in multipotent hematopoietic progenitors. J. Exp. Med..

[bib41] Lauridsen F.K.B., Jensen T.L., Rapin N., Aslan D., Wilhelmson A.S., Pundhir S., Rehn M., Paul F., Giladi A., Hasemann M.S. (2018). Differences in Cell Cycle Status Underlie Transcriptional Heterogeneity in the HSC Compartment. Cell Rep..

[bib42] Leentjens J., Bekkering S., Joosten L.A.B., Netea M.G., Burgner D.P., Riksen N.P. (2018). Trained Innate Immunity as a Novel Mechanism Linking Infection and the Development of Atherosclerosis. Circ. Res..

[bib43] Lewis K.N., Liao R., Guinn K.M., Hickey M.J., Smith S., Behr M.A., Sherman D.R. (2003). Deletion of RD1 from Mycobacterium tuberculosis Mimics Bacille Calmette-Guérin Attenuation. J. Infect. Dis..

[bib44] Liberzon A., Birger C., Thorvaldsdottir H., Ghandi M., Mesirov J.P., Tamayo P. (2015). The Molecular Signatures Database (MSigDB) hallmark gene set collection. Cell Syst..

[bib45] Lun A.T., McCarthy D.J., Marioni J.C. (2016). A step-by-step workflow for low-level analysis of single-cell RNA-seq data with Bioconductor. F1000Res..

[bib46] Marcela Rodriguez G., Neyrolles O. (2014). Metallobiology of Tuberculosis. Microbiol. Spectr..

[bib47] Martin-Sanchez D., Gallegos-Villalobos A., Fontecha-Barriuso M., Carrasco S., Sanchez-Nino M.D., Lopez-Hernandez F.J., Ruiz-Ortega M., Egido J., Ortiz A., Sanz A.B. (2017). Deferasirox-induced iron depletion promotes BclxL downregulation and death of proximal tubular cells. Sci. Rep..

[bib48] Matatall K.A., Jeong M., Chen S., Sun D., Chen F., Mo Q., Kimmel M., King K.Y. (2016). Chronic Infection Depletes Hematopoietic Stem Cells through Stress-Induced Terminal Differentiation. Cell Rep..

[bib49] Mayer-Barber K.D., Andrade B.B., Oland S.D., Amaral E.P., Barber D.L., Gonzales J., Derrick S.C., Shi R., Kumar N.P., Wei W. (2014). Host-directed therapy of tuberculosis based on interleukin-1 and type I interferon crosstalk. Nature.

[bib50] Mitroulis I., Ruppova K., Wang B., Chen L.S., Grzybek M., Grinenko T., Eugster A., Troullinaki M., Palladini A., Kourtzelis I. (2018). Modulation of Myelopoiesis Progenitors Is an Integral Component of Trained Immunity. Cell.

[bib51] Moorlag S., Khan N., Novakovic B., Kaufmann E., Jansen T., van Crevel R., Divangahi M., Netea M.G. (2020). beta-Glucan Induces Protective Trained Immunity against Mycobacterium tuberculosis Infection: A Key Role for IL-1. Cell Rep..

[bib52] Murray M.J., Murray A.B., Murray M.B., Murray C.J. (1978). The adverse effect of iron repletion on the course of certain infections. BMJ.

[bib53] Muto Y., Nishiyama M., Nita A., Moroishi T., Nakayama K.I. (2017). Essential role of FBXL5-mediated cellular iron homeostasis in maintenance of hematopoietic stem cells. Nat. Commun..

[bib54] Nandi B., Behar S.M. (2011). Regulation of neutrophils by interferon-gamma limits lung inflammation during tuberculosis infection. J. Exp. Med..

[bib55] Nemes E., Geldenhuys H., Rozot V., Rutkowski K.T., Ratangee F., Bilek N., Mabwe S., Makhethe L., Erasmus M., Toefy A. (2018). Prevention of M. tuberculosis Infection with H4:IC31 Vaccine or BCG Revaccination. N. Engl. J. Med..

[bib56] Netea M.G., Domínguez-Andrés J., Barreiro L.B., Chavakis T., Divangahi M., Fuchs E., Joosten L.A.B., van der Meer J.W.M., Mhlanga M.M., Mulder W.J.M. (2020). Defining trained immunity and its role in health and disease. Nat. Rev. Immunol..

[bib57] Newton K., Dugger D.L., Wickliffe K.E., Kapoor N., de Almagro M.C., Vucic D., Komuves L., Ferrando R.E., French D.M., Webster J. (2014). Activity of protein kinase RIPK3 determines whether cells die by necroptosis or apoptosis. Science.

[bib58] Novershtern N., Subramanian A., Lawton L.N., Mak R.H., Haining W.N., McConkey M.E., Habib N., Yosef N., Chang C.Y., Shay T. (2011). Densely interconnected transcriptional circuits control cell states in human hematopoiesis. Cell.

[bib59] Olakanmi O., Schlesinger L.S., Britigan B.E. (2007). Hereditary hemochromatosis results in decreased iron acquisition and growth by Mycobacterium tuberculosis within human macrophages. J. Leukoc. Biol..

[bib60] Parrow N.L., Fleming R.E., Minnick M.F. (2013). Sequestration and scavenging of iron in infection. Infect. Immun..

[bib61] Paul F., Arkin Y., Giladi A., Jaitin D.A., Kenigsberg E., Keren-Shaul H., Winter D., Lara-Astiaso D., Gury M., Weiner A. (2015). Transcriptional Heterogeneity and Lineage Commitment in Myeloid Progenitors. Cell.

[bib62] Pietras E.M., Lakshminarasimhan R., Techner J.M., Fong S., Flach J., Binnewies M., Passegue E. (2014). Re-entry into quiescence protects hematopoietic stem cells from the killing effect of chronic exposure to type I interferons. J. Exp. Med..

[bib63] Platanias L.C., Fish E.N. (1999). Signaling pathways activated by interferons. Exp. Hematol..

[bib64] Polager S., Ginsberg D. (2009). p53 and E2f: partners in life and death. Nat. Rev. Cancer.

[bib65] Reddy P.V., Puri R.V., Khera A., Tyagi A.K. (2012). Iron storage proteins are essential for the survival and pathogenesis of Mycobacterium tuberculosis in THP-1 macrophages and the guinea pig model of infection. J. Bacteriol..

[bib66] Reddy V.P., Chinta K.C., Saini V., Glasgow J.N., Hull T.D., Traylor A., Rey-Stolle F., Soares M.P., Madansein R., Rahman M.A. (2018). Ferritin H Deficiency in Myeloid Compartments Dysregulates Host Energy Metabolism and Increases Susceptibility to Mycobacterium tuberculosis Infection. Front. Immunol..

[bib67] Regev D., Surolia R., Karki S., Zolak J., Montes-Worboys A., Oliva O., Guroji P., Saini V., Steyn A.J., Agarwal A. (2012). Heme oxygenase-1 promotes granuloma development and protects against dissemination of mycobacteria. Lab. Invest..

[bib68] Rickard J.A., O’Donnell J.A., Evans J.M., Lalaoui N., Poh A.R., Rogers T., Vince J.E., Lawlor K.E., Ninnis R.L., Anderton H. (2014). RIPK1 regulates RIPK3-MLKL-driven systemic inflammation and emergency hematopoiesis. Cell.

[bib69] Ritchie M.E., Phipson B., Wu D., Hu Y., Law C.W., Shi W., Smyth G.K. (2015). limma powers differential expression analyses for RNA-sequencing and microarray studies. Nucleic Acids Res..

[bib70] Robinson M.D., McCarthy D.J., Smyth G.K. (2010). edgeR: a Bioconductor package for differential expression analysis of digital gene expression data. Bioinformatics.

[bib71] Roca F.J., Ramakrishnan L. (2013). TNF dually mediates resistance and susceptibility to mycobacteria via mitochondrial reactive oxygen species. Cell.

[bib72] Roderick J.E., Hermance N., Zelic M., Simmons M.J., Polykratis A., Pasparakis M., Kelliher M.A. (2014). Hematopoietic RIPK1 deficiency results in bone marrow failure caused by apoptosis and RIPK3-mediated necroptosis. Proc. Natl. Acad. Sci. USA.

[bib73] Rodrigo T., Cayla J.A., Garcia de Olalla P., Galdos-Tanguis H., Jansa J.M., Miranda P., Brugal T. (1997). Characteristics of tuberculosis patients who generate secondary cases. Int. J. Tuberc. Lung Dis..

[bib74] Samstein M., Schreiber H.A., Leiner I.M., Susac B., Glickman M.S., Pamer E.G. (2013). Essential yet limited role for CCR2(+) inflammatory monocytes during Mycobacterium tuberculosis-specific T cell priming. eLife.

[bib75] Satija R., Farrell J.A., Gennert D., Schier A.F., Regev A. (2015). Spatial reconstruction of single-cell gene expression data. Nat. Biotechnol..

[bib76] Sato T., Onai N., Yoshihara H., Arai F., Suda T., Ohteki T. (2009). Interferon regulatory factor-2 protects quiescent hematopoietic stem cells from type I interferon-dependent exhaustion. Nat. Med..

[bib77] Shannon P., Markiel A., Ozier O., Baliga N.S., Wang J.T., Ramage D., Amin N., Schwikowski B., Ideker T. (2003). Cytoscape: a software environment for integrated models of biomolecular interaction networks. Genome Res..

[bib78] Sherman D.R., Guinn K.M., Hickey M.J., Mathur S.K., Zakel K.L., Smith S. (2004). Mycobacterium tuberculosis H37Rv:ΔRD1 Is More Virulent than M. bovis Bacille Calmette-Guérin in Long-Term Murine Infection. J. Infect. Dis..

[bib79] Silva-Gomes S., Appelberg R., Larsen R., Soares M.P., Gomes M.S. (2013). Heme catabolism by heme oxygenase-1 confers host resistance to Mycobacterium infection. Infect. Immun..

[bib80] Smith J.N.P., Zhang Y., Li J.J., McCabe A., Jo H.J., Maloney J., MacNamara K.C. (2018). Type I IFNs drive hematopoietic stem and progenitor cell collapse via impaired proliferation and increased RIPK1-dependent cell death during shock-like ehrlichial infection. PLoS Pathog..

[bib81] Soares M.P., Weiss G. (2015). The Iron age of host-microbe interactions. EMBO Rep..

[bib82] Stuart T., Butler A., Hoffman P., Hafemeister C., Papalexi E., Mauck W.M., Hao Y., Stoeckius M., Smibert P., Satija R. (2019). Comprehensive Integration of Single-Cell Data. Cell.

[bib83] Tameris M.D., Hatherill M., Landry B.S., Scriba T.J., Snowden M.A., Lockhart S., Shea J.E., McClain J.B., Hussey G.D., Hanekom W.A. (2013). Safety and efficacy of MVA85A, a new tuberculosis vaccine, in infants previously vaccinated with BCG: a randomised, placebo-controlled phase 2b trial. Lancet.

[bib84] Tzelepis F., Blagih J., Khan N., Gillard J., Mendonca L., Roy D.G., Ma E.H., Joubert P., Jones R.G., Divangahi M. (2018). Mitochondrial cyclophilin D regulates T cell metabolic responses and disease tolerance to tuberculosis. Sci. Immunol..

[bib85] Vandenabeele P., Galluzzi L., Vanden Berghe T., Kroemer G. (2010). Molecular mechanisms of necroptosis: an ordered cellular explosion. Nat. Rev. Mol. Cell Biol..

[bib86] Wendeln A.C., Degenhardt K., Kaurani L., Gertig M., Ulas T., Jain G., Wagner J., Hasler L.M., Wild K., Skodras A. (2018). Innate immune memory in the brain shapes neurological disease hallmarks. Nature.

[bib87] Wolf A.J., Desvignes L., Linas B., Banaiee N., Tamura T., Takatsu K., Ernst J.D. (2008). Initiation of the adaptive immune response to Mycobacterium tuberculosis depends on antigen production in the local lymph node, not the lungs. J. Exp. Med..

[bib88] Yamashita M., Passegue E. (2019). TNF-alpha Coordinates Hematopoietic Stem Cell Survival and Myeloid Regeneration. Cell Stem Cell.

[bib89] Zhao X., Khan N., Gan H., Tzelepis F., Nishimura T., Park S.Y., Divangahi M., Remold H.G. (2017). Bcl-xL mediates RIPK3-dependent necrosis in M. tuberculosis-infected macrophages. Mucosal Immunol..

[bib90] Zheng G.X.Y., Terry J.M., Belgrader P., Ryvkin P., Bent Z.W., Wilson R., Ziraldo S.B., Wheeler T.D., McDermott G.P., Zhu J. (2017). Massively parallel digital transcriptional profiling of single cells. Nat. Commun..

